# Different stigmas, different patterns: unfair treatment maps onto bullying involvement through internalizing and externalizing problems

**DOI:** 10.3389/fpsyg.2026.1780810

**Published:** 2026-06-18

**Authors:** Xiaoran Zhang, Jinxiao Ginnie Liu, Wanjing Li, Chengyu Xu, Ruiqi Deng, Wailon Chong

**Affiliations:** 1 School of Public Administration & Law, Fujian Agriculture and Forestry University, Fuzhou, China; 2 Faculty of Law, University of Macau, Taipa, Macao SAR, China; 3 Faculty of Humanities and Social Sciences, City University of Macau, Macao, Macao SAR, China; 4 Faculty of Education, City University of Macau, Macao, Macao SAR, China

**Keywords:** bullying perpetration, bullying victimization, externalizing problems, internalizing problems, unfair treatment

## Abstract

**Background:**

Bias-based bullying is often treated as a single problem, yet different prejudices may show different associations with distinct psychological roles. With many jurisdictions lacking protections for bias, identifying which forms of stigma have the strongest connections with both bullying victimization and bullying perpetration is urgent.

**Methods:**

We analyzed the 2022 National Survey of Children's Health using comparative multiple-mediator association models linking racial/ethnic, disability-related, and sexual orientation or gender identity-based unfair treatment to bullying victimization and bullying perpetration via internalizing and externalizing problems. The model used percentage-scale coefficients, with 5,000 bootstrap samples, and pathway coefficients were compared to evaluate whether association patterns differed across types of unfair treatment.

**Results:**

All three unfair-treatment indicators were positively associated with bullying victimization. In the primary unweighted models, only sexual orientation or gender identity-based unfair treatment retained a direct association with perpetration after accounting for internalizing and externalizing problems. Indirect statistical associations via internalizing were observed for all three unfair-treatment indicators for both outcomes, whereas the externalizing route was evident only for sexual orientation or gender identity-based unfair treatment. Pairwise comparisons suggested larger sexual orientation or gender identity-based coefficients than racial/ethnic- or disability-related coefficients in several pathways.

**Conclusion:**

Distinct prejudices correspond to distinct psychological association patterns. Sexual orientation or gender identity-based unfair treatment is associated with internal distress with outward dysregulation, as well as greater involvement in both bullying roles. Findings suggest the potential value of precision prevention: universal protections plus enumerated sexual orientation or gender identity safeguards and affirming climates, with targeted support for internalizing burdens among racially minoritized and disabled youth. Comparative coefficient testing helps identify where these associations appear relatively stronger.

## Introduction

Bullying in adolescence is a pervasive form of interpersonal aggression associated with substantial and enduring harm. It is typically defined as intentional, repeated aggressive behavior among peers that exploits a power imbalance and leaves targets unable to defend themselves (Volk et al., 2014). Such behavior can be physical, verbal, relational, sexual, or cyber in nature and constitutes a serious global public health concern. Recent data underline its alarming prevalence: international surveys suggest that roughly one third of adolescents worldwide have been subjected to bullying at school, and even in high-income settings the burden remains substantial, with about 34% of U.S. teenagers reporting being bullied within a single year (Koyanagi et al., 2019; Li et al., 2020). Bullied children and adolescents are nearly three times as likely to develop depression as their uninvolved peers and around twice as likely to report suicidal ideation or attempts (van Geel et al., 2014; Ye et al., 2023). These figures are not merely abstract statistics but reflect adolescents' lived experiences; they correspond to millions of young people whose everyday lives are marked by fear, humiliation, and disrupted development.

Being targeted for an immutable, core aspect of identity can be particularly traumatic: prejudice-driven mistreatment related to race/ethnicity, disability, or sexual orientation or gender identity can impose unique and cumulative burdens on young people (Benner et al., 2018; Russell and Fish, 2016). Recent national surveillance data indicate that about one in three U.S. high-school students have experienced racism in school (McKinnon, 2024). The burden is heaviest for Asian, multiracial, and Black students; for example, nearly 60% of Asian students report experiencing racism at school. Among Black and Hispanic adolescents, those who report racism show consistently higher rates of poor mental health, suicide risk, and substance use than same-race peers who do not report racism. Parallel challenges confront young people with disabilities. A global review led by UNESCO finds that children with disabilities are three to four times more likely than their non-disabled peers to experience violence and up to twice as likely to be bullied at school (Hughes et al., 2012; Jones et al., 2012). Sexual and gender minority youth face similarly elevated levels of bias-based harassment and bullying victimization in more recent studies (Fish et al., 2023; Pérez-Albéniz et al., 2025; Webb et al., 2021). These adolescents also bear a disproportionate burden of emotional distress, substance use, and suicidal behavior (Marshal et al., 2011; Poštuvan et al., 2019). Taken together, the evidence underscores the urgency of supporting young people exposed to unfair treatment in disengaging from bullying dynamics and interrupting this self-perpetuating cycle of harm. Here, we use unfair treatment to refer broadly to identity-related experiences of being treated or judged unfairly, whether or not they occur within bullying episodes. By contrast, bias-based bullying refers to bullying that explicitly targets a stigmatized identity, whereas bullying victimization and bullying perpetration refer to the more general bullying outcomes measured in the NSCH and are not themselves coded as bias-based.

Bias-based bullying can precipitate profound emotional distress (internalizing outcomes) while also engendering anger, distrust, or dysregulated behavior (externalizing outcomes) in youth (Reijntjes et al., 2010; Ye et al., 2023). Minority stress, stress-process/general strain, social information processing, and developmental psychopathology perspectives all suggest that bias-based aggression and exclusion communicate devaluation, which some children internalize as low self-worth and psychological turmoil and others externalize as reactive aggression or bullying of others (Crick and Dodge, 1994; Russell and Fish, 2016). In our conceptual model, internalizing and externalizing problems, in line with prior work, are collectively referred to as psychological ill-being (Liu et al., 2024), and are treated as explanatory variables that may help account for associations between unfair treatment and later bullying involvement (Fisher et al., 2016). In a recent cross-sectional study of 3,939 high-school students, adolescents who reported identity-based bullying had nearly threefold higher odds of non-suicidal self-injury and suicidal ideation, as well as roughly two- to fivefold higher odds of multiple forms of violence involvement, including bullying perpetration, compared with peers who reported no such bullying. These patterns suggest that identity-based bullying may be embedded in a broader cycle of peer harm. Being targeted because of a stigmatized or marginalized identity can heighten social threat, humiliation, exclusion, and psychological distress, thereby increasing vulnerability to self-directed harm. Repeated exposure to hostile peer environments may also foster retaliatory aggression, defensive dominance behaviors, or involvement in coercive peer interactions, making it possible for adolescents to occupy both victim and perpetrator roles. Although cross-sectional evidence cannot establish temporal sequencing, the clustering of victimization, self-harm risk, and violence involvement is consistent with a vicious-cycle framework in which bullying victimization contributes to psychological and interpersonal strain, and these strains may increase the likelihood of further bullying involvement. This logic underpins the present study's rationale: bullying should be understood as a recursive social process that can sustain both internalizing harm and continued peer aggression (Galán et al., 2021).

What remains uncertain, however, is whether different forms of prejudice exert distinct influences on internalizing vs. externalizing trajectories (Benner et al., 2018). Does racial discrimination primarily instill anxiety and depression, whereas anti-LGBTQ+ victimization more strongly provokes anger and acting out? Or are the psychological sequelae of various biases largely similar? To date, the literature has not conclusively answered these questions: many studies focus on a single type of bias, and few have directly compared how racism, ableism, and homophobia differentially shape adolescents' emotional and behavioral responses. Clarifying these nuances is crucial for tailoring interventions, as it would help determine whether particular marginalized groups are at greater risk of inward suffering or outward aggression in the face of mistreatment.

To address these gaps, the present study adopts a methodological approach that uses coefficient comparison techniques to examine whether associations differ across multiple direct and indirect statistical paths (MacKinnon, 2012; Preacher and Hayes, 2008). We use this framework to compare association patterns linking racial/ethnic, disability-related, and sexual orientation or gender identity-related unfair treatment to adolescent internalizing and externalizing outcomes (Hayes, 2017; VanderWeele, 2015). Minority stress and related developmental perspectives further suggest that stigma types may differ in visibility, concealability, chronicity, and available coping resources, making distinct internalizing vs. externalizing association profiles theoretically plausible (Meyer, 2003; Pachankis and Clark, 2025). By quantifying and comparing the strength of these pathways on a common effect-size scale, the study aims to determine which forms of unfair treatment show relatively stronger associations with different outcomes and explanatory variables. This comparative framework offers descriptive evidence on how inequality may be linked to psychosocial difficulties and bullying involvement among marginalized adolescents.

## Literature review and hypotheses development

**Figure 1:**
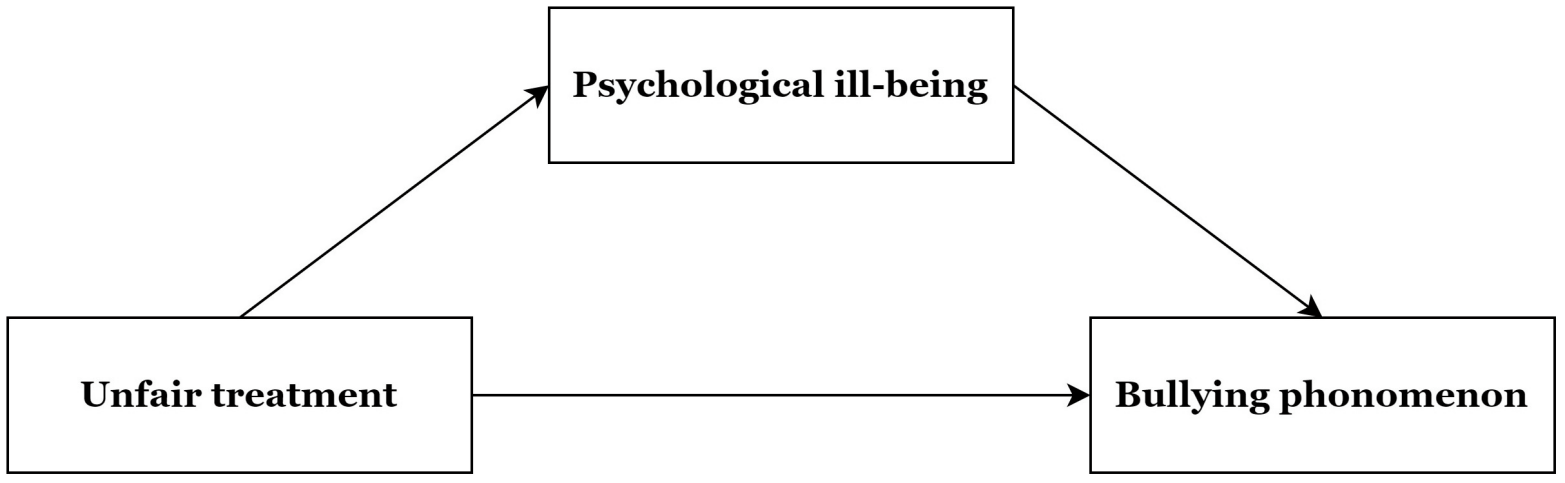
Conceptual map of research subject.

Different types of unfair treatment may be associated with distinct patterns of mental health issues among children, warranting separate consideration of racial/ethnic, sexual orientation or gender identity-related, and disability-related unfair treatment. Experiences of unfair treatment are often linked to children's psychological wellbeing, manifesting predominantly as internalizing and externalizing problems (Cho and Lee, 2018; Juvonen and Graham, 2014). Internalizing problems primarily encompass adverse emotional states such as anxiety and depression (Achenbach, 1966), whereas externalizing problems involve behaviors directed outward that negatively impact the social environment, including aggression and conduct disorders (Eisenberg et al., 2001). Given the differing meanings and measurement of bullying involvement, we distinguish bullying victimization from bullying perpetration throughout the study. To enhance conceptual clarity and facilitate subsequent analysis, this paper proposes a conceptual map illustrated in Figure 1.

### Identity-based unfair treatment as a driver of psychological ill-being

Research on mental and physical health consistently indicates that early-life mental illness is predictive of adverse health outcomes later in life (Najman et al., 2022). For instance, internalizing problems during adolescence significantly predict more severe anxiety and depressive disorders in middle adulthood (Herrenkohl et al., 2010). People, who are exposed to a variety of stressors, face higher rates of risk of internalizing problems, especially elevated anxiety and depressive symptoms (Liu et al., 2016). For children and adolescents, exposure to external stressors, such as perceived teacher's unfairness or negative contact from the police (Gini et al., 2018; Nivette et al., 2024), it is associated with a negative emotional response, which may persist long after the encounter, ultimately harming mental health and wellbeing (Testa et al., 2021). Equally, externalizing problems are prevalent in contrast to internalizing problems. Setbacks on the winding road to adulthood can be stressful and can also lead to increased risk-taking and externalizing problems (Bos et al., 2021).

Firstly, racial discrimination (e.g., overt exclusion, microaggressions) increases children's psychological ill-being. Racial/ethnic discrimination is among the best-characterized forms of unfair treatment in child development, with strong evidence for internalizing sequelae across diverse settings (Benner et al., 2018). In an urban cohort of African American youth followed from grades 7–10 (*n* = 504), those in an increasing-discrimination trajectory were four times more likely to show an increasing depression trajectory than peers with stable-low exposure (Smith-Bynum et al., 2014). Physiological embedding is documented: discrimination assessed from adolescence predicted flatter diurnal cortisol slopes and a lower cortisol awakening response 20 years later (Adam et al., 2015). While mentally, perceived devaluation of one's racial identity fosters internalized stigma and rumination, which exacerbate feelings of worthlessness and social withdrawal. Regarding externalizing problems, persistent discrimination generates anger and hostility, which manifest as aggression toward peers or authority figures.

Secondly, unfair treatment related to sexual orientation or gender identity (SOGI) is commonly framed by minority-stress models, which posit that stigma-related stressors amplify psychopathology through cognitive, affective, and interpersonal pathways. In syntheses of the evidence, internalizing outcomes dominate: a systematic review reported a pooled depression prevalence of 26% among sexual and gender minority young people and repeatedly highlighted victimization/harassment as a key risk factor (O'Shea et al., 2025). Longitudinal data strengthen inference: in a two-wave study of 197 adolescents, sexual-minority-specific victimization significantly mediated disparities in depressive symptoms and suicidality over six months (Burton et al., 2013). These patterns align with stress vigilance and emotion dysregulation processes. For externalizing consequences, in a community cohort of young sexual minority men, concurrent victimization covaried with higher externalizing symptoms and substance use (Swann et al., 2019). Furthermore, targeted youth may engage in verbal/physical aggression to assert control in hostile environments, particularly when protective factors (e.g., school support) are lacking.

Thirdly, disability-related unfair treatment has been less systematically examined than racism or SOGI stigma, yet the available evidence points to a comparatively consistent internalizing burden. A recent systematic review concluded that unfair treatment by disability is linked to psychological distress, depression, anxiety, suicidality, and diminished self-confidence and outlook (Lindsay et al., 2025). Repeated devaluation is incorporated into self-concept and erodes belonging even in the absence of overt harassment (Jóhannsdóttir et al., 2022). By contrast, the evidence for externalizing problems is thinner and often indirect: in a three-wave, two-year study of 441 families of autistic children, parents' discrimination predicted later child externalizing as well as internalizing via caregiver depression, harsh parenting, and coparenting conflict (Chan et al., 2023). In addition, repeated denial of accommodations (e.g., inaccessible spaces) causes sensory overload and emotional dysregulation, triggering impulsive aggression.

Scholars have argued that unfair treatment of minorities is particularly detrimental to the mental health and psychological adjustment of adolescents, as it can undermine their identity development (Balkaya et al., 2019). Minorities learn how others perceive their group and how social rewards, punishments, stressors, resources, and justice are distributed accordingly, and end up with positive or negative feelings about the whole group, leading to corresponding psychological consequences (Foy et al., 2014; Hughes et al., 2016; Pearlin and Bierman, 2013; Thoits, 2010).

Based on the above, this study examined the following hypothesis:

**H1a:** Experiencing racial unfair treatment will be positively associated with children's psychological ill-being, manifesting in both internalizing and externalizing problems.
**H1b:** Experiencing unfair treatment related to sexual orientation or gender identity will be positively associated with children's psychological ill-being, manifesting in both internalizing and externalizing problems.
**H1c:** Experiencing disability-related unfair treatment will be positively associated with children's psychological ill-being, manifesting in both internalizing and externalizing problems.

### Psychological ill-being as predictors of bullying involvement

In youth mental health frameworks, mental health is often conceptualized to include both positive functioning (psychological wellbeing) and negative functioning (psychological ill-being). In line with prior work in JAMA Pediatrics, we use psychological ill-being to refer to internalizing and externalizing problems (Liu et al., 2024). Both are relevant to bullying involvement, although not necessarily in the same way. There is burgeoning literature on internalizing problems and bullying involvement. For example, (Finnegan et al. 1996) believed that psychological difficulties may also precede subsequent victimization. Child victims often display internalized behaviors, such as fear and social withdrawal, which can make them more vulnerable to bullying victimization by peers (Reijntjes et al., 2010). Moreover, their internalizing problems may also hamper their ability to form supportive friendships that might otherwise protect them from bullying victimization (Reijntjes et al., 2010). In fact, bullying perpetration resulting from internalizing problems is equally prevalent compared to bullying victimization. In particular, adolescents, who have experienced more victimization, are more likely to engage in perpetration either in self-defense or due to aggressive tendencies caused by frequent victimization (Sousa et al., 2024). Studies have shown that children with internalizing problems see bullying perpetration as a maladaptive coping mechanism that temporarily alleviates feelings of insecurity and social exclusion (Georgiou et al., 2013).

As for externalizing problems, children often have difficulty controlling impulses, and such difficulties increase the likelihood of participating in bullying dynamics, leading to conflict with peers and social exclusion (Sentse et al., 2015), increasing their chances of being targeted (Pouwels et al., 2016b). For example, teens with ADHD symptoms reported higher rates of bullying victimization (Chen et al., 2020). On the other hand, adolescents with externalizing problems are more likely to have trouble interpreting social cues and coping with complex interactions, which could explain their increased involvement in bullying perpetration (de Sousa et al., 2021). They use aggression to handle social interactions, especially when they feel threatened or feel the need to exercise power over others (Pouwels et al., 2016a).

Both internalizing and externalizing symptoms are problematic because their adverse effects are not only immediate but also long-term (Quintana-Orts et al., 2023). Some research conceptualizes children's psychological wellbeing as the extent to which children experience these two types of problems (Chen and Liu, 2012; Evans and De France, 2022). Internalizing and externalizing problems are therefore expected to be associated with both bullying victimization and bullying perpetration.

Based on the above, this study proposed the following hypothesis:

1. **H2a:** Children's internalizing problems will be positively associated with bullying involvement, manifesting in both bullying victimization and perpetration.
2. **H2b:** Children's externalizing problems will be positively associated with bullying involvement, manifesting in both bullying victimization and perpetration.

### Indirect association framework from unfair treatment to bullying involvement

Prior theory and empirical work suggest that unfair treatment may be associated with bullying victimization and perpetration partly through psychological ill-being (Jinxiao Ginnie et al., 2026). Empirical evidence increasingly identifies perceived unfair treatment as a catalyst for bullying perpetration through psychological ill-being. Studies reveal that chronic exposure to unfair treatment (e.g., teacher favoritism, peer exclusion) fosters depressive symptoms and social anxiety, which distort interpersonal perceptions. The above situation leads adolescents to interpret ambiguous social behaviors as hostile are 2.1 times more likely to preemptively bully peers to avoid perceived victimization (Leadbeater et al., 2022; Nikiforou et al., 2013). Neurobiological evidence further indicates that cortisol dysregulation from prolonged unfairness amplifies amygdala reactivity, predisposing youth to impulsive aggression (McLaughlin et al., 2019). Meta-analyses show that adolescents with ADHD symptoms display 3.4-fold higher rates of proactive bullying when subjected to unfair parental treatment (Klemp et al., 2024; Larzelere et al., 2020). Experimental studies verify that unfair treatment trigger externalizing youths' dominance-driven bullying, with 42% justifying aggression as “restoring fairness” (Pouwels et al., 2016b).

Unfair treatment heightens the possibility of bullying victimization by eroding psychological resilience. Internalizing pathways are evident in correlations between perceived discrimination and social withdrawal: youth reporting high school-based injustice exhibit 1.8-fold greater withdrawal behaviors (Sirin et al., 2015), which peers often misinterpret as weakness, triggering being targeted (Sentse et al., 2015). (Galand and Hospel 2013)'study revealed that internalizing symptoms mediate 31% of the linkage between unfair teacher treatment and peer victimization. Externalizing pathways might operate through peer rejection dynamics. (Kjellstrand et al. 2018)'study demonstrated that children rated high in externalizing behaviors due to unfair familial practices faced 67% higher odds of victimization, as peers sanctioned their disruptive conduct. These youth also tend to misread social cues, inadvertently escalating conflicts—empirical work shows people with externalizing problems misunderstand a lot of peaceful peer intentions as hostile, doubling victimization risk (Fung, 2019).

Accordingly, the present study treats internalizing and externalizing problems as explanatory variables that may partly account for associations between unfair treatment and bullying involvement. Ultimately highlighting the dual function of children's psychological ill-being because of being treated unfairly and a predictor of bullying phenomenon.

Based on the above, this study proposed the following hypothesis:

1. **H3:** Associations between unfair treatment and bullying involvement will be partly accounted for by internalizing and externalizing problems.

## Research methodology

### Data and study population

The study is based on the National Survey of Children's Health (NSCH) funded and supervised by the Health Resources and Services Administration (HRSA) and Maternal and Child Health Bureau (MCHB). The NSCH is designed to provide comprehensive data on the physical and mental health of children aged 0–17 across the United States, which uses a meticulously crafted cross-sectional approach to ensure representativeness across diverse demographics and regions. In 2022, it successfully surveyed 54,103 households with children aged 0–17 years, representing the non-institutional population between the ages of 0–17 at both national and state levels. More information about the 2022 NSCH can be found elsewhere (Bureau, 2025).

Because the bullying items are asked of adolescents, the present analyses focused on youths ages 12–17. Model 1 (bullying victimization) included 30,400 participants (14,591 female and 15,809 male), and Model 2 (bullying perpetration) included 30,389 participants (14,582 female and 15,807 male).

### Measurement

The core variables, including three items of treated unfairly (race, sexual orientation or gender identity, and disability), two internalizing problems items (anxiety and depression), two externalizing problems items (ADHD and behavioral problems), and bullying phenomenon (bullied and bullying), along with other covariates, were extracted from the 2022 NSCH dataset.

### Treated unfairly

The 2022 NSCH provides information on three forms of unfair treatment: race/ethnicity, sexual orientation or gender identity, and health condition and disability, as reported by the parents under the section “I. About Your Family and Household”. Namely that, has this child EVER experienced any of the following (1) Treated or judged unfairly because of their race or ethnic group; (2) Treated or judged unfairly because of their sexual orientation or gender identity; (3) Treated or judged unfairly because of a health condition or disability. The responses were binary (yes or no), and it is worth noting that the cumulative score reflects a higher exposure to adversity (Bethell et al., 2017).

### Externalizing problems

Externalizing problems was assessed using two items from the 2022 NSCH: “Has a doctor, other health care provider or educator EVER told you that this child has (1) Behavioral or conduct problems? (2) Attention Deficit Disorder or Attention-Deficit/Hyperactivity Disorder, that is, ADD or ADHD?” The responses were binary (yes or no). We recoded these items and created a composite measure by summing the two responses, ensuring that higher scores reflect a greater degree of externalizing problems (Khanijahani and Sualp, 2022).

### Internalizing problems

The 2022 NSCH included two items measuring internalizing problems, asking: “Has a doctor or other health care provider EVER told you that this child has (1) Anxiety problems? (2) Depression?” The optional answers to these items are binary yes or no. We recoded these items and summed up the two responses so that the higher the score, the higher the degree of internalizing problems (Karalunas et al., 2023; Khanijahani and Sualp, 2022).

### Bullying perpetration

In 2022, NSCH started a project to study the variable of bullying perpetration, and asked the following questions: During the past 12 months, how often did this child bully others, pick on them, or exclude them? Do not include siblings or dating partners. If the frequency changed throughout the year, report the highest frequency. There are five types of answers. 1 = Never (in the past 12 months), 2 = 1–2 times (in the past 12 months), 3 = 1–2 times per month, 4 = 1–2 times per week, and 5 = Almost every day, with higher scores therefore indicating more frequent bullying perpetration (Haegele et al., 2020; Lebrun-Harris et al., 2019).

### Bullying victimization

The 2022 NSCH asked the following questions to study the variable of bullying victimization: During the past 12 months, how often was this child bullied, picked on, or excluded by other children? Do not include siblings or dating partners. If the frequency changed throughout the year, report the highest frequency. There are five types of answers. 1 = Never (in the past 12 months), 2 = 1–2 times (in the past 12 months), 3 = 1–2 times per month, 4 = 1–2 times per week, and 5 = Almost every day, with higher scores therefore indicating more frequent bullying victimization (Haegele et al., 2020; Lebrun-Harris et al., 2019).

### Explanatory covariates

To control for potential confounders that may affect the relationship between unfair treatment, externalizing problems, internalizing problems, bullying perpetration, and bullying victimization, several covariates were included in our analyses in addition to the main variables. These included the child's sex, the mother's age at the child's birth, the caregiver's sex, the caregiver's employment, the caregiver's physical health and mental health, the caregiver's relationship with selected child, whether the caregiver was born in the U.S., household income as a percentage of the Federal Poverty Level (FPL), and the number of family members. These were all reported by the children's parents. Established guidelines for good controls were followed in the selection of control variables for this study. These controls were chosen to reduce confounding (Cinelli et al., 2024; Gelman et al., 2021; Rubin, 2009). These covariates were included in all reported models. Reference groups in the regression tables were selected to facilitate descriptive comparison within the sample.

### Data analysis strategy

Descriptive statistics for all study variables were obtained using Python 3.12. The hypotheses were then evaluated by estimating regression models with PROCESS v5.3 in R (Hayes, 2017; Lyu et al., 2022). Both unstandardized coefficients (β) and percentage-scale coefficients (*b*_*p*_) were presented, following prior recommendations. The *b*_*p*_ metric reflects the percentage change in the outcome for a full-scale (0 → 1) increase in the predictor, thereby providing an intuitive effect size that facilitates comparison across variables and outcomes with different scales (Zhao et al., 2025, 2024). This approach has been widely adopted and applied in multiple fields such as public health, communications, and marketing (Ao et al., 2023; Luo and Homburg, 2008; Zhang et al., 2024; Zhu et al., 2025). Employing *b*_*p*_ addresses persistent critiques of standardized βs in cross-model comparisons, given that standard deviations are sample- and context-dependent and may lead to distorted interpretations when distributions vary significantly between groups (Blalock Jr, 1961; King, 1986; Li et al., 2025a).

Although bullying victimization and bullying perpetration were measured as 5-category ordinal frequency variables, the primary analyses modeled them as ordered frequency scores so that all paths could be estimated on a common coefficient scale for *b*_*p*_ estimation and coefficient comparison. A fuller methodological rationale, including the tradeoffs relative to ordered logistic models, is provided in Supplementary Note 1.

In addition, pairwise comparisons of *b*_*p*_ coefficients were performed to test for statistically significant differences in effect sizes between predictors. Specifically, differences in absolute magnitude (|*b*_*p*_ (*i*)|–| *b*_*p*_ (*j*)|) was examined, with statistical significance evaluated via percentile bootstrap confidence intervals (Zhao et al., 2025, 2024). Computational details for these comparisons can be found in the two aforementioned articles.

Indirect effects were quantified by averaging the products of the relevant *b*_*p*_ coefficients across 5,000 bias-corrected bootstrap samples. This approach has been shown to generate stable estimates and confidence intervals for indirect statistical associations (Hayes, 2017; Zhao et al., 2010). Complete syntax and detailed examples for implementing *b*_*p*_ estimation and the bootstrap procedure in both Python and the PROCESS Macro can be found in the online appendices of (Han et al. 2023) and (Zhao et al. 2024).

Because PROCESS v5.3 does not directly accommodate NSCH sampling weights or design-based variance estimation, the primary mediation models should be interpreted as unweighted association models. We therefore conducted supplementary weighted robustness analyses in R using lavaan with NSCH sampling weights and Huber-White heteroskedasticity-robust standard errors; these analyses broadly supported the main conclusions, and full results are reported in Supplementary Tables 1–5 and Supplementary Note 2.

As an additional sensitivity analysis, we estimated Bayesian directional-plausibility models to compare the hypothesized ordering with selected reverse-order alternatives. These checks were used only to rank relative directional compatibility rather than to establish causality; fuller details are provided in Supplementary Note 3 and Supplementary Tables 10–12.

## Results

### Demographics of this study

**Figure 2:**
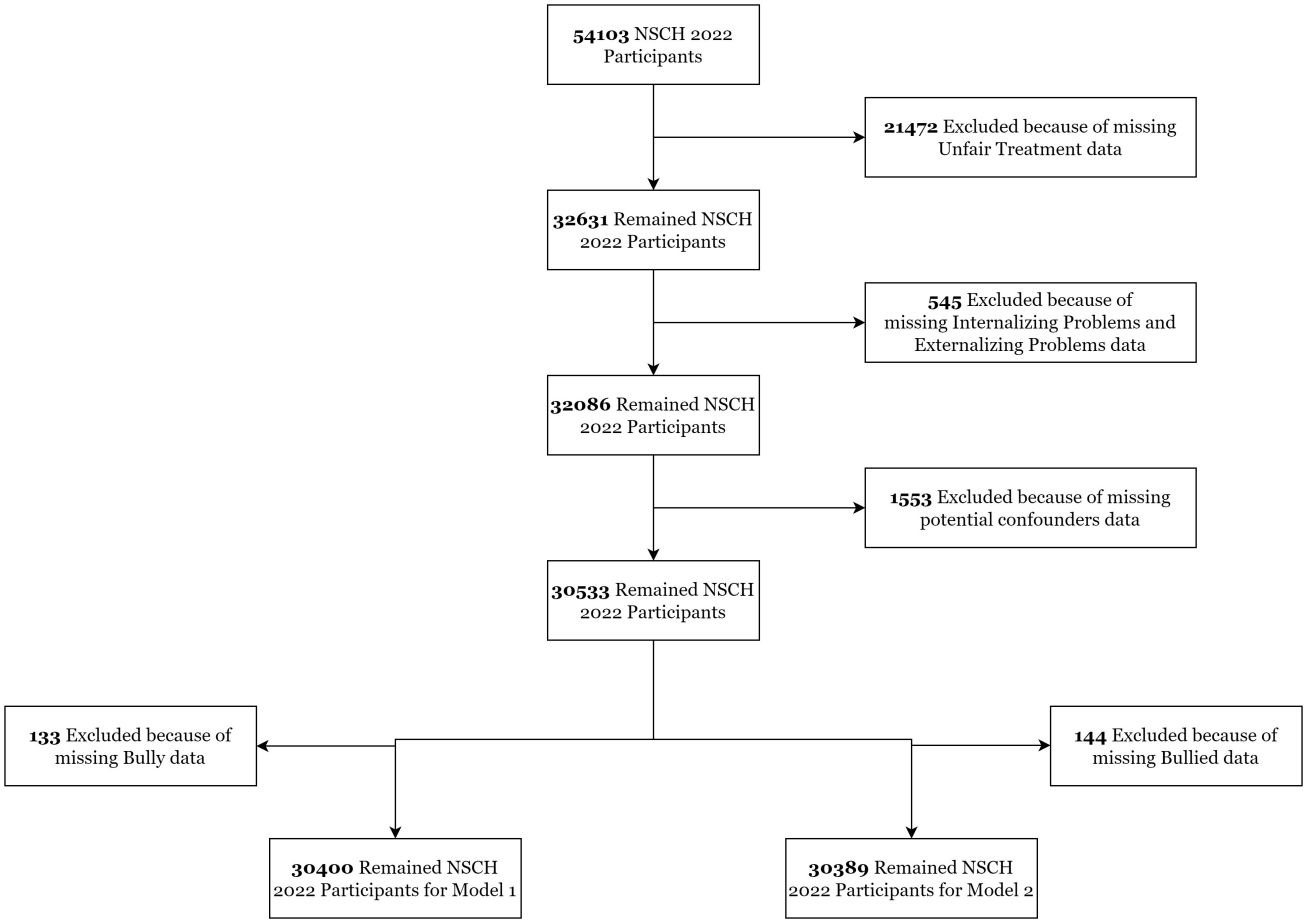
Flowchart of study participant selection.

A total of approximately 30,400 children were included in model 1 and 30,389 in Model 2 (see Table 1). After excluding cases with missing data (see Figure 2 for participant flow), the final analytic sample comprised roughly mid-adolescent youths (ages 12–17). The mean age was around 14 years, and about half of participants were female. The sample was predominantly non-Hispanic White (65.61%), followed by Hispanic (14.90–14.92%), non-Hispanic multiracial (7.65–7.66%), non-Hispanic Black (5.98–6.00%), and non-Hispanic Asian (5.84%). Caregiver-reported unfair treatment due to race/ethnicity was reported for 4.74% of adolescents, SOGI-related unfair treatment for 4.39%, and disability-related unfair treatment for 2.27%. Mean bullying victimization was 1.65 in Model 1, and mean bullying perpetration was 1.21 in Model 2. These prevalence rates align with national estimates and underscore that a substantial subset of youths were exposed to bullying and bias-related adversity.

**Table 1:** Frequency and proportion table of samples from Model 1 and Model 2.

Characteristic	Model 1	Model 2
*N*	30400	30389
Unfair treatment by race	1440 (4.74%)	1440 (4.74%)
Unfair treatment by sexual orientation or gender identity	1334 (4.39%)	1334 (4.39%)
15.6-8,-1.3243ptUnfair treatment by disability	691 (2.27%)	689 (2.27%)
Selected child's sex		
Female	14591 (48.00%)	14582 (47.98%)
Male	15809 (52.00%)	15807 (52.02%)
15.6-8,-1.3243ptMom's age (mean)	30.20	30.20
Selected child's race		
Hispanic	4537 (14.92%)	4529 (14.90%)
Non-Hispanic Asian	1773 (5.84%)	1775 (5.84%)
Non-Hispanic Black	1818 (5.98%)	1822 (6.00%)
Non-Hispanic Multirace	2328 (7.66%)	2326 (7.65%)
15.6-8,-1.3243ptNon-Hispanic White	19944 (65.61%)	19937 (65.61%)
Caregiver sex		
Female	20891 (68.72%)	20888 (68.74%)
15.6-8,-1.3243ptMale	9509 (31.28%)	9501 (31.26%)
Caregiver's employment		
Employed full-time	21092(69.38%)	21087 (69.39%)
Employed part-time	3699 (12.17%)	3690 (12.14%)
Working without pay	392 (13.11%)	392 (1.29%)
Not employed but looking for work	1232 (4.05%)	1230 (4.05%)v
Not employed and not looking for work	3985 (13.11%)	3990 (13.13%)
Caregiver's physical health (mean)	2.12	2.12
29-8,0243ptCaregiver's mental health (mean)	2.20	2.20
Household income as a percentage of the Federal Poverty level		
0–99% FPL	3548 (11.67%)	3538 (11.64%)
100–199% FPL	4894 (16.10%)	4892 (16.10%)
200–399% FPL	8995 (29.59%)	8999 (29.61%)
15.6-8,-1.3243pt400% FPL or greater	12963 (42.64%)	12960 (42.65%)
Number of family members in 2022		
1 Member	2332 (7.67%)	2332 (7.67%)
2 Members	8515 (28.01%)	8508 (28.00%)
3 Members	11558 (38.02%)	11562 (38.05%)
4 Members	5409 (17.80%)	5409 (17.80%)
15.6-8,-1.3243pt5 Members	2586 (8.51%)	2578 (8.48%)
Caregiver's relationship with selected child		
Biological or adoptive parent	27732 (91.22%)	27719 (91.21%)
Step-parent	757 (2.49%)	755 (2.48%)
Foster parent	58 (0.19%)	58 (0.19%)
Grand parent	1414 (4.65%)	1416 (4.66%)
Other: non-relative	345 (1.13%)	348 (1.15%)
Other: relative	94 (0.31%)	93 (0.31%)
Caregiver born in the U.S.		
Yes	25677 (84.46%)	25671 (84.47%)
No	4723 (15.54%)	4718 (15.53%)
Internalizing problems	2281 (7.50%)	2282 (7.51%)
Externalizing problems	2165 (7.12%)	2164 (7.12%)
Bullying victimization (mean)	1.64	1.65
Bullying perpetration (mean)	1.21	1.21

### Multiple mediation analysis

**Figure 3:**
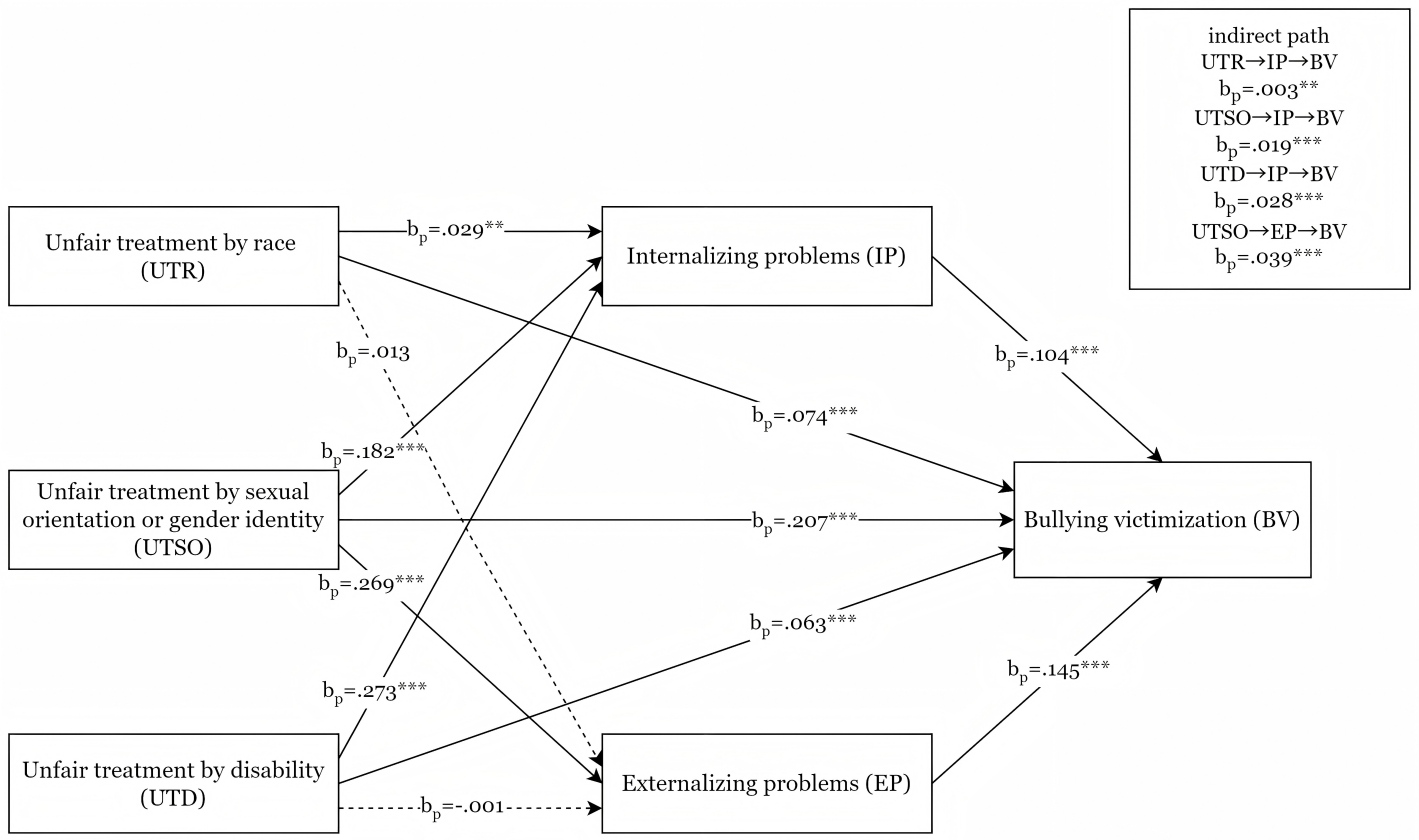
Multiple-mediator association model for bullying victimization. bp, percentage coefficient; UTR, unfair treatment by race; UTSO, unfair treatment by sexual orientation or gender identity. UTD, unfair treatment disability; IP, internalizing problems; EP, externalizing problems; BV, Bullying victimization, *p < 0.05; **p < 0.01; ***p < 0.001.

Table 2 presents the regression results for bullying victimization (Model 1). In Model 1 (see Figure 3), all three unfair treatment indicators showed significant positive associations with bullying victimization, supporting H1a–H1c. Specifically, adolescents who had ever been treated unfairly due to their race/ethnicity were more likely to report past-year victimization (*b*_*p*_ = 0.074^***^, β = 0.296^***^). SOGI-related unfair treatment showed the largest positive direct association with bullying victimization (*b*_*p*_ = 0.207^***^, β = 0.827^***^), and unfair treatment due to a health condition or disability was also positively associated with elevated odds of victimization (*b*_*p*_ = 0.063^***^, β = 0.252^***^). In addition, both indices of psychological ill-being were significant independent predictors of victimization, consistent with H2. Youth with higher internalizing problems had greater risk of being bullied (*b*_*p*_ = 0.104^***^, β = 0.579^***^), and those with more externalizing problems were also more likely to be victimized (*b*_*p*_ = 0.145^***^, β = 0.417^***^). Notably, the magnitude of the externalizing-victimization link slightly exceeded that of internalizing in this model, suggesting that youth behavioral difficulties may attract peer bullying to at least a similar extent as emotional distress does.

**Table 2:** Direct associations in Model 1 (bullying victimization).

Predictor	Internalizing problems		Externalizing problems		Bullying victimization	
	*b_*p*_*	β	*b_*p*_*	β	*b_*p*_*	β
Unfair treatment by race	0.029^**^	0.029^**^	0.013	0.014	0.074^***^	0.296^***^
Unfair treatment by sexual orientation or gender identity	0.182^***^	0.182^***^	0.269^***^	0.269^***^	0.207^***^	0.827^***^
Unfair treatment by disability	0.273^***^	0.273^***^	−0.001	−0.0003	0.063^***^	0.252^***^
Selected child's sex (1 = Female)	0.040^***^	0.040^***^	−0.057^***^	−0.057^***^	0.011^***^	0.044^***^
15.6-8,-1.3500ptMom's age	−0.036^***^	−0.001^***^	−0.057^***^	−0.002^***^	−0.012^*^	−0.002^*^
Selected child's race (reference “Black”)						
Hispanic	0.043^***^	0.043^***^	0.007	0.007	0.017^**^	0.069^**^
White	0.055^***^	0.055^***^	0.022^**^	0.022^**^	0.053^***^	0.213^***^
Asian	0.027^***^	0.027^***^	−0.004	−0.004	0.007	0.028
Multiracial	0.045^***^	0.046^***^	0.015	0.016^*^	0.025^***^	0.098^***^
15.6-8,-1.3500ptCaregiver sex (1 = Female)	0.020^***^	0.020^***^	0.004	0.004	0.023^***^	0.092^***^
Caregiver's employment (reference “Not employed and not looking for work”)						
Employed full-time	−0.007	−0.008	−0.010^*^	−0.011^*^	0.011^**^	0.044^**^
Employed part-time	−0.015^*^	−0.015^*^	−0.016^**^	−0.016^**^	0.006	0.024
Employed without pay	0.002	0.002	−0.016	−0.017	0.017	0.070
Not employed but looking for work	−0.005	−0.005	−0.006	−0.006	0.014	0.054
Caregiver's physical health	−0.115^***^	−0.029^***^	−0.091^***^	−0.023^***^	−0.125^***^	−0.125^***^
Caregiver's mental health	−0.035^***^	−0.009^***^	−0.030^***^	−0.008^***^	−0.030^***^	−0.030^***^
Household income as a percentage of the federal poverty level	−0.012^**^	−0.0001^**^	−0.015^**^	−0.0001^**^	0.013^***^	0.0001^**^
Number of family members in 2022	−0.045^***^	−0.011^***^	−0.033^***^	−0.008^***^	−0.005	−0.005
Caregiver's relationship with selected child (reference “Other: Relative”)						
Biological or adoptive parent	−0.035	−0.034	0.0001	−0.001	−0.037	−0.146
Step-parent	−0.003	−0.003	0.031	0.030	−0.017	−0.066
Foster parent	0.106	0.108	0.213^***^	0.213^**^	0.0001	0.005
Grand parent	−0.009	−0.009	0.046	0.045	−0.026	−0.099
Other: non-relative	0.006	0.005	0.042	0.041	−0.075^**^	−0.297^**^
Caregiver born in the U.S. (1 = outside the U.S.)	−0.013^***^	−0.013^**^	−0.015^***^	−0.015^***^	−0.028^***^	−0.112^***^
Internalizing problems					0.104^***^	0.579^***^
Externalizing problems					0.145^***^	0.417^***^
*N*	30389		30389		30389	
*R*-squared	0.102		0.093		0.189	
Adj *R*-squared	0.101		0.093		0.188	

*b*_*p*_, percentage coefficient (Zhao et al., 2024); β, unstandardized coefficient. Coefficients and significance levels were estimated using percentile bootstrap following (Zhao et al. 2010).

^*^p < 0.05, ^**^p < 0.01, ^***^p < 0.001.

**Figure 4:**
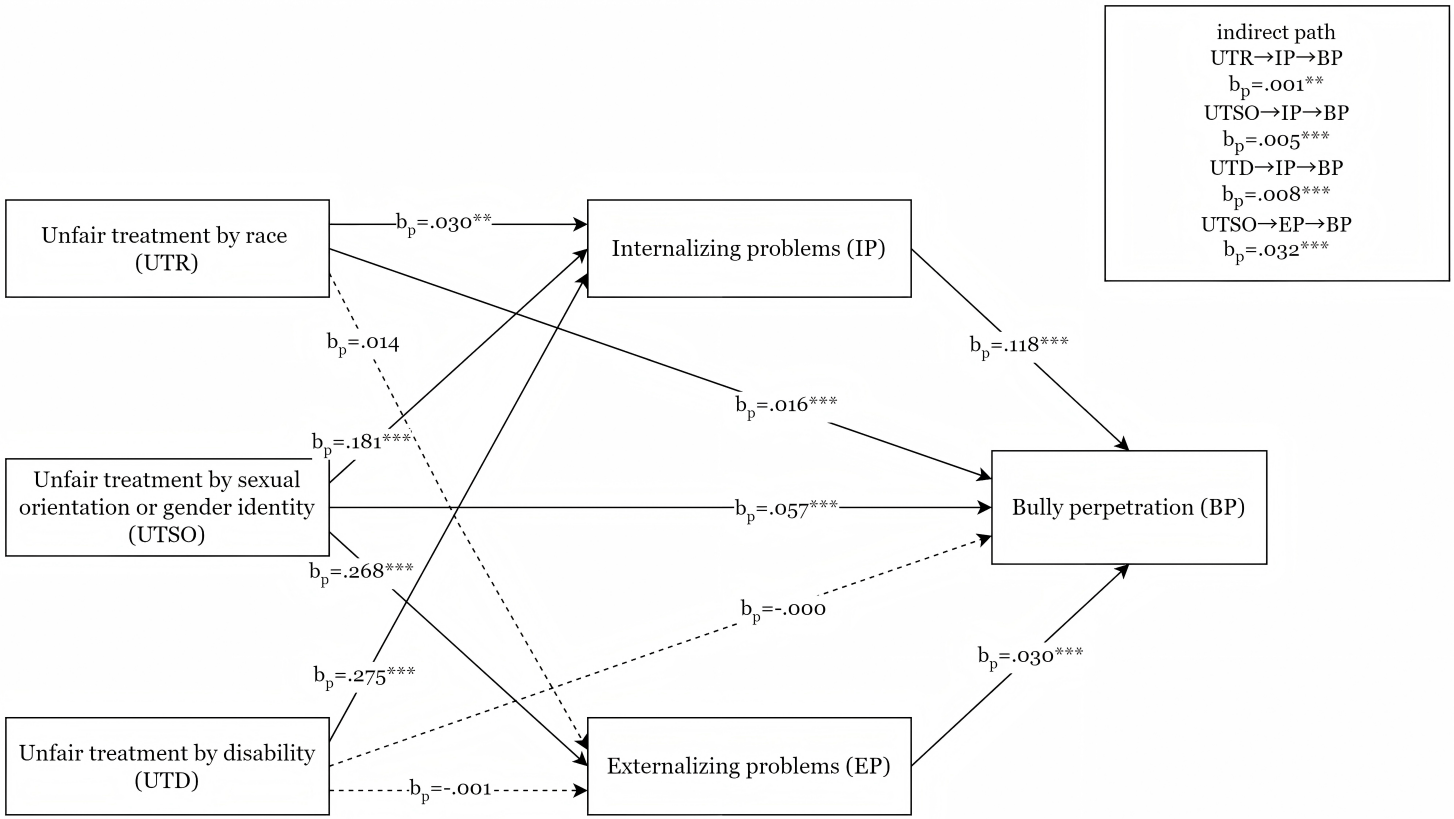
Multiple-mediator association model for bullying perpetration. bp, percentage coefficient; UTR, unfair treatment by race; UTSO, unfair treatment by sexual orientation or gender identity. UTD, unfair treatment disability; IP, internalizing problems; EP, externalizing problems; BV, Bullying victimization, *p < 0.05; **p < 0.01; ***p < 0.001.

Table 3 displays the direct associations for bullying perpetration (Model 2). In Model 2 (see Figure 4), only one form of unfair treatment maintained a significant unique positive association with bullying perpetration: SOGI-related unfair treatment (*b*_*p*_ = 0.057^***^, β = 0.228^***^). By contrast, race-based and disability-based unfair treatment were not significantly associated with bullying perpetration after accounting for psychological ill-being (*p* > 0.05), indicating that H1a and H1c were not supported for the perpetration after controlling for mediators. In line with H2, psychological ill-being remained an important predictor of perpetration: internalizing problems showed a positive association with bullying perpetration (*b*_*p*_ = 0.118^***^, β = 0.471^***^), whereas externalizing problems had a smaller yet significant positive association (*b*_*p*_ = 0.030^***^, β = 0.118^***^). In supplementary survey-weighted robustness analyses, the overall pattern remained similar, but the weighted perpetration model detected a small positive direct association with race-based unfair treatment and yielded a larger externalizing than internalizing coefficient. We therefore avoid strong claims about the relative dominance of internalizing vs. externalizing problems for perpetration. The most stable conclusion across specifications is that SOGI-related unfair treatment remained the clearest direct correlate of perpetration, whereas both dimensions of psychological ill-being were relevant to perpetration.

**Table 3:** Direct associations in Model 2 (bullying perpetration).

Predictor	Internalizing problems		Externalizing problems		Bullying perpetration	
	*b_*p*_*	β	*b_*p*_*	β	*b_*p*_*	β
Unfair treatment by race	0.030^**^	0.030^**^	0.014	0.014	0.016^***^	0.063^**^
Unfair treatment by sexual orientation or gender identity	0.181^***^	0.181^***^	0.268^***^	0.268^***^	0.057^***^	0.228^***^
Unfair treatment by disability	0.275^***^	0.275^***^	−0.001	−0.001	−0.0001	0.001
Selected child's sex (1 = female)	0.040^***^	0.040^***^	−0.057^***^	−0.057^***^	−0.004^**^	−0.016^**^
Mom's age	−0.036^***^	−0.001^***^	−0.057^***^	−0.002^***^	−0.019^***^	−0.003^***^
Selected child's race (reference “Black”)						
Hispanic	0.043^***^	0.043^***^	0.006	0.006	0.004	0.015
White	0.056^***^	0.056^***^	0.021^**^	0.021^***^	0.017^***^	0.067^***^
Asian	0.027^***^	0.027^***^	−0.004	−0.004	0.001	0.005
Multiracial	0.046^***^	0.046^***^	0.015	0.015	0.009^*^	0.035^*^
Caregiver sex (1 = female)	0.020^***^	0.020^***^	0.004	0.004	0.009^***^	0.036^***^
Caregiver's employment (reference “Not employed and not looking for work”)						
Employed full-time	−0.008	−0.008	−0.011^*^	−0.011^*^	0.011^***^	0.042^***^
Employed part-time	−0.014^*^	−0.014^*^	−0.017^**^	−0.017^**^	0.007^*^	0.029^*^
Employed without pay	0.002	0.002	−0.016	−0.016	0.007	0.028
Not employed but looking for work	−0.004	−0.004	−0.007	−0.007	0.003	0.010
Caregiver's physical health	−0.114^***^	−0.029^***^	−0.091^***^	−0.023^***^	−0.061^***^	−0.061^***^
Caregiver's mental health	−0.035^***^	−0.009^***^	−0.030^***^	−0.008^***^	0.004	0.004
Household income as a percentage of the federal poverty level	−0.012^**^	−0.0001^**^	−0.015^**^	−0.0001^**^	0.001	0.0001
Number of family members in 2022	−0.045^***^	−0.011^***^	−0.033^***^	−0.008^***^	0.002	0.002
Caregiver's relationship with selected child (reference “Other: relative”)						
Biological or adoptive parent	−0.033	−0.033	0.0003	0.0004	−0.042^*^	−0.170^*^
Step-parent	−0.001	−0.001	0.031	0.031	0.005	0.019
Foster parent	0.109	0.110	0.211^**^	0.215^**^	0.082	0.334
Grand parent	−0.007	−0.007	0.047	0.047	−0.033	−0.132
Other: non-relative	0.007	0.006	0.039	0.039	−0.041^*^	−0.166^*^
Caregiver born in the U.S. (1 = outside the U.S.)	−0.013^**^	−0.013^**^	−0.015^***^	−0.015^***^	−0.017^***^	−0.067^***^
Internalizing problems					0.118^***^	0.471^***^
Externalizing problems					0.030^***^	0.118^***^
*N*	30400		30400		30400	
*R*-squared	0.102		0.093		0.116	
Adj *R*-squared	0.101		0.092		0.115	

*b*_*p*_, percentage coefficient (Zhao et al., 2024); β, unstandardized coefficient. Coefficients and significance levels were estimated using percentile bootstrap following (Zhao et al. 2010).

^*^p < 0.05, ^**^p < 0.01, ^***^p < 0.001.

The multiple-mediator models were consistent with indirect association patterns linking unfair treatment, psychological ill-being, and bullying involvement, supporting H3. As shown in the path diagrams of Figures 3, 4 (and detailed in Tables 4, 5), youths who faced unfair treatment tended to exhibit higher psychological ill-being, which were in turn statistically associated with bullying outcomes.

**Table 4:** Indirect associations in Model 1 (bullying victimization).

Indirect path	*b_*p*_*	β	95% CI
UTR → IP → BV	0.003^**^	0.012^**^	0.001, 0.005
UTSO → IP → BV	0.019^***^	0.076^***^	0.016, 0.023
UTD → IP → BV	0.028^***^	0.113^***^	0.023, 0.034
UTR → EP → BV	0.002	0.008	−0.0004, 0.004
UTSO → EP → BV	0.039^***^	0.156^***^	0.034, 0.044
UTD → EP → BV	−0.0001	−0.0002	−0.004, 0.004

*b*_*p*_, percentage coefficient; β, unstandardized coefficient; UTR, unfair treatment by race; UTSO, unfair treatment by sexual orientation or gender identity; UTD, unfair treatment by disability; IP, internalizing problems; EP, externalizing problems; BV, bullying victimization.

^*^p < 0.05, ^**^p < 0.01, ^***^p < 0.001.

**Table 5:** Indirect associations in Model 2 (bullying perpetration).

Indirect path	*b_*p*_*	β	95% CI
UTR → IP → BP	0.001^**^	0.004^**^	0.0003, 0.002
UTSO → IP → BP	0.005^***^	0.021^***^	0.004, 0.007
UTD → IP → BP	0.008^***^	0.032^***^	0.006, 0.012
UTR → EP → BP	0.002	0.007	−0.0003, 0.004
UTSO → EP → BP	0.032^***^	0.126^***^	0.028, 0.036
UTD → EP → BP	−0.0001	−0.0003	−0.003, 0.003

*b*_*p*_, percentage coefficient; β, unstandardized coefficient; UTR, unfair treatment by race; UTSO, unfair treatment by sexual orientation or gender identity; UTD, unfair treatment by disability; IP, internalizing problems; EP, externalizing problems; BP, bullying perpetration.

^*^p < 0.05, ^**^p < 0.01, ^***^p < 0.001.

In Model 1 (victimization), significant indirect statistical associations via internalizing problems were observed for unfair treatment due to race/ethnicity (*b*_*p*_ = 0.003^**^ when transmitted through internalizing, indicating a small yet reliable mediated effect), sexual orientation or gender identity (*b*_*p*_ = 0.019^***^ via internalizing), and disability (*b*_*p*_ = 0.028^***^ via internalizing). In other words, these unfair-treatment indicators were associated with higher internalizing symptom levels, and internalizing problems were positively associated with bullying victimization. Additionally, SOGI-related unfair treatment yielded a significant indirect statistical association via externalizing problems in the victimization model (*b*_*p*_ = 0.039^***^), reflecting that this form of discrimination also fostered behavioral problems that made youth more susceptible to victimization. The corresponding externalizing routes for race- and disability-related unfair treatment were not significant (consistent with the non-significant direct paths from those discrimination types to externalizing in Figure 3).

In Model 2 (perpetration), a similar pattern emerged: all three unfair-treatment indicators showed small but significant statistical associations with bullying perpetration through internalizing problems (*b*_*p*_ = 0.001^**^ for race, 0.005^***^ for sexual orientation or gender identity, and 0.008^***^ for disability). Only SOGI-related unfair treatment showed a significant indirect statistical association via externalizing problems on perpetration (*b*_*p*_ = 0.032^***^). This pattern is consistent with a stronger externalizing-linked association in the SOGI-related models. Neither race- nor disability-related unfair treatment exhibited a meaningful indirect association through externalizing in the perpetration model (as their associations with externalizing were near zero). Overall, the results were consistent with indirect statistical association patterns in which psychological ill-being, particularly internalizing problems, was linked to both bullying outcomes. These findings are depicted in Figures 3, 4, which highlight the significant pathways (solid arrows) for each model.

### Comparative magnitude of associations

We compared associations using scalar differences in efficiencies (*d*_*s*_ = |*b*_*p*_ (*i*)|–| *b*_*p*_ (*j*)|) with percentile-bootstrap inference; matrices follow the recommended *b*_*p*_ format, enabling like-for-like contrasts on a common 0–1 scale (Tables 6–9; *b*_*p*_ and unstandardized β are in Tables 2–5).

**Table 6:** Scalar comparison of efficiencies—direct paths of Model 1.

path(*j*) path(*i*)	UTR → IP	UTSO → IP	UTD → IP	UTR → EP	UTSO → EP	UTD → EP	UTR → BV	UTSO → BV	UTD → BV	EP → BV	IP → BV
UTR → IP	—	0.153^***^	0.244^***^	−0.016	0.240^***^	−0.019	0.045^***^	0.177^***^	0.033^*^	0.075^***^	0.116^***^
UTSO → IP	−0.153^***^	—	0.091^***^	−0.168^***^	0.087^***^	−0.172^***^	−0.108^***^	0.025	−0.119^***^	−0.078^***^	−0.037^**^
UTD → IP	−0.244^***^	−0.091^***^	—	−0.259^***^	−0.004	−0.263^***^	−0.199^***^	−0.066^***^	−0.210^***^	−0.169^***^	−0.128^***^
UTR → EP	0.016	0.168^***^	0.259^***^	—	0.255^***^	−0.004	0.060^***^	0.193^***^	0.049^***^	0.091^***^	0.131^***^
UTSO → EP	−0.240^***^	−0.087^***^	0.004	−0.255^***^	—	−0.259^***^	−0.195^***^	−0.063^***^	−0.207^***^	−0.165^***^	−0.124^***^
UTD → EP	0.019	0.172^***^	0.263^***^	0.004	0.259^***^	—	0.064^***^	0.197^***^	0.053^***^	0.094^***^	0.135^***^
UTR → BV	−0.045^***^	0.108^***^	0.199^***^	−0.060^***^	0.195^***^	−0.064^***^	—	0.133^***^	−0.011	0.030^**^	0.071^***^
UTSO → BV	−0.177^***^	−0.025	0.066^***^	−0.193^***^	0.063^***^	−0.197^***^	−0.133^***^	—	−0.144^***^	−0.102^***^	−0.062^***^
UTD → BV	−0.033^*^	0.119^***^	0.210^***^	−0.049^***^	0.207^***^	−0.053^***^	0.011	0.144^***^	—	0.042^**^	0.082^***^
EP → BV	−0.075^***^	0.078^***^	0.169^***^	−0.091^***^	0.165^***^	−0.094^***^	−0.030^**^	0.102^***^	−0.042^**^	—	0.041^***^
IP → BV	−0.116^***^	0.037^**^	0.128^***^	−0.131^***^	0.124^***^	−0.135^***^	−0.071^***^	0.062^***^	−0.082^***^	−0.041^***^	—

Main cells show scalar differences between efficiencies (d_s_ = |b_p_(i)|–|b_p_(j)|). Diagonal entries are em dashes (—). ^*^ *p* < 0.05, ^**^ *p* < 0.01, ^***^ *p* < 0.001.

UTR, unfair treatment by race; UTSO, unfair treatment by sexual orientation or gender identity; UTD, unfair treatment by disability; IP, internalizing problems; EP, externalizing problems; BV, bullying victimization.

**Table 7:** Scalar comparison of efficiencies—indirect paths of Model 1.

path(*j*) path(*i*)	UTR → IP → BV	UTSO → IP → BV	UTD → IP → BV	UTR → EP → BV	UTSO → EP → BV	UTD → EP → BV
UTR → IP → BV	—	0.016^***^	0.025^***^	−0.001	0.036^***^	−0.002
UTSO → IP → BV	−0.016^***^	—	0.010^***^	−0.017^***^	0.020^***^	−0.018^***^
UTD → IP → BV	−0.025^***^	−0.010^***^	—	−0.027^***^	0.011^**^	−0.027^***^
UTR → EP → BV	0.001	0.017^***^	0.027^***^	—	0.037^***^	−0.001
UTSO → EP → BV	−0.036^***^	−0.020^***^	−0.011^**^	−0.037^***^	—	−0.038^***^
UTD → EP → BV	0.002	0.018^***^	0.027^***^	0.001	0.038^***^	—

Main cells show scalar differences between efficiencies (d_s_ = |b_p_(i)|–|b_p_(j)|). Diagonal entries are em dashes (—). ^*^ *p* < 0.05, ^**^ *p* < 0.01, ^***^ *p* < 0.001.

UTR, unfair treatment by race; UTSO, unfair treatment by sexual orientation or gender identity; UTD, unfair treatment by disability; IP, internalizing problems; EP, externalizing problems; BV, bullying victimization.

**Table 8:** Scalar comparison of efficiencies—direct paths of Model 2.

path(*j*) path(*i*)	UTR → IP	UTD → IP	UTSO → IP	UTR → EP	UTD → EP	UTSO → EP	UTR → BP	UTD → BP	UTSO → BP	EP → BP	IP → BP
UTR → IP	—	0.244^***^	0.152^***^	−0.016	−0.020	0.238^***^	−0.014	−0.024^*^	0.028^*^	0.088^***^	−0.0001
UTD → IP	−0.245^***^	—	−0.094^***^	−0.261^***^	−0.264^***^	−0.007	−0.259^***^	−0.269^***^	−0.217^***^	−0.157^***^	−0.245^***^
UTSO → IP	−0.152^***^	0.094^***^	—	−0.167^***^	−0.171^***^	0.087^***^	−0.165^***^	−0.175^***^	−0.124^***^	−0.063^***^	−0.152^***^
UTR → EP	0.016	0.261^***^	0.167^***^	—	−0.004	0.254^***^	0.002	−0.008	0.043^***^	0.104^***^	0.016
UTD → EP	0.020	0.264^***^	0.171^***^	0.004	—	0.258^***^	0.006	−0.004	0.047^***^	0.108^***^	0.019
UTSO → EP	−0.238^***^	0.007	−0.087^***^	−0.254^***^	−0.258^***^	—	−0.252^***^	−0.262^***^	−0.211^***^	−0.150^***^	−0.238^***^
UTR → BP	0.014	0.259^***^	0.165^***^	−0.002	−0.006	0.252^***^	—	−0.010	0.041^***^	0.102^***^	0.014^*^
UTD → BP	0.024^*^	0.269^***^	0.175^***^	0.008	0.004	0.262^***^	0.010	—	0.051^***^	0.112^***^	0.024^***^
UTSO → BP	−0.028^*^	0.217^***^	0.124^***^	−0.043^***^	−0.047^***^	0.211^***^	−0.041^***^	−0.051^***^	—	0.061^***^	−0.028^***^
EP → BP	−0.088^***^	0.157^***^	0.063^***^	−0.104^***^	−0.108^***^	0.150^***^	−0.102^***^	−0.112^***^	−0.061^***^	—	−0.088^***^
IP → BP	0.0001	0.245^***^	0.152^***^	−0.016	−0.019	0.238^***^	−0.014^*^	−0.024^***^	0.028^***^	0.088^***^	—

Main cells show scalar differences between efficiencies (d_s_ = |b_p_(i)|–|b_p_(j)|). Diagonal entries are em dashes (—). ^*^ *p* < 0.05, ^**^ *p* < 0.01, ^***^ *p* < 0.001.

UTR, unfair treatment by race; UTSO, unfair treatment by sexual orientation or gender identity; UTD, unfair treatment by disability; IP, internalizing problems; EP, externalizing problems; BP, bullying perpetration.

**Table 9:** Scalar comparison of efficiencies—indirect paths of Model 2.

path(*j*) path(*i*)	UTR → IP → BP	UTD → IP → BP	UTSO → IP → BP	UTR → EP → BP	UTD → EP → BP	UTSO → EP → BP
UTR → IP → BP	—	0.007^***^	0.005^***^	0.001	0.0003	0.031^***^
UTD → IP → BP	−0.007^***^	—	−0.003^***^	−0.007^***^	−0.007^***^	0.023^***^
UTSO → IP → BP	−0.005^***^	0.003^***^	—	−0.004^**^	−0.004^**^	0.026^***^
UTR → EP → BP	−0.001	0.007^***^	0.004^**^	—	−0.001	0.030^***^
UTD → EP → BP	−0.0003	0.007^***^	0.004^**^	0.001	—	0.030^***^
UTSO → EP → BP	−0.031^***^	−0.023^***^	−0.026^***^	−0.030^***^	−0.030^***^	—

Main cells show scalar differences between efficiencies (d_s_ = |b_p_(i)|–|b_p_(j)|). Diagonal entries are em dashes (—). ^*^ *p* < 0.05, ^**^ *p* < 0.01, ^***^ *p* < 0.001.

UTR, unfair treatment by race; UTSO, unfair treatment by sexual orientation or gender identity; UTD, unfair treatment by disability; IP, internalizing problems; EP, externalizing problems; BP, bullying perpetration.

In the coefficient-comparison matrices (Tables 6–9), SOGI-related unfair treatment generally showed the most pronounced direct and indirect coefficients, especially along externalizing-linked routes. In Model 1, the direct victimization coefficient for SOGI-related unfair treatment exceeded the corresponding race- and disability-related coefficients (vs. race: *d*_*s*_ = 0.133^***^; vs. disability: *d*_*s*_ = 0.144^***^), and the largest indirect victimization route was the SOGI-externalizing path. In Model 2, the direct perpetration coefficient for SOGI-related unfair treatment again exceeded race and disability (vs race: *d*_*s*_ = 0.041^***^; vs. disability: *d*_*s*_ = 0.051^***^), and the largest indirect route was likewise the SOGI-externalizing path. Because the weighted perpetration robustness model did not preserve the same internalizing-vs.-externalizing rank order, we avoid treating that endpoint ordering as definitive. These *d*_*s*_ results therefore most strongly reinforce H1b and H3, while providing more qualified support for H1a/H1c and H2 across outcomes. A fuller narrative walkthrough of Tables 6–9 is provided in Supplementary Note 4. Survey-weighted scalar-comparison matrices corresponding to these contrasts are reported in Supplementary Tables 6–9.

Because the three unfair-treatment indicators can co-occur within the same youth, we conducted supplementary weighted interaction analyses and SHAP-based overlap checks. These exploratory analyses suggested that the highest predicted burden on both bullying victimization and bullying perpetration occurred under triple exposure, consistent with cumulative disadvantage under multiple unfair-treatment domains. At the same time, several pairwise interaction terms were negative, indicating that overlap was not uniformly additive on the observed outcome scale. Because the predictive fit of the exploratory perpetration interaction model was limited, these findings should be interpreted as descriptive evidence that co-occurrence matters. Fuller results and interpretation are provided in Supplementary Note 5, Supplementary Tables 13–15, and Supplementary Figures 3–5. Weighted SHAP importance profiles are shown in Supplementary Figures 1, 2.

## Discussion

### Main findings

Our findings indicate that race-based, sexual orientation or gender identity-based, and disability-based unfair treatment were each associated with bullying involvement, but the pattern of association differed across discrimination types (Benner et al., 2018; Cave et al., 2020). Race-based unfair treatment was more closely associated with outwardly directed difficulties, whereas sexual orientation or gender identity-based unfair treatment showed a stronger association with internalizing problems and bullying victimization (Oshri et al., 2024; Toomey and Anhalt, 2016). Disability-based unfair treatment was linked to both pathways, suggesting a broader vulnerability in peer contexts (Augustine et al., 2024; Pinquart, 2017). These findings are consistent with prior work showing that bias-based victimization can be especially consequential because it targets socially devalued aspects of the self, thereby compounding the harms associated with more generalized peer conflict (Galán et al., 2021; Mulvey et al., 2018).

These contrasts also warrant cautious interpretation. One possibility is that different forms of unfair treatment vary in the social meanings they carry and therefore in the ways they are psychologically processed. Sexual orientation or gender identity-based unfair treatment may be especially likely to intensify inward distress because it can threaten identity coherence while fostering concealment, anticipated rejection, and fear of disclosure (Meyer, 2003; Pérez-Albéniz et al., 2025; Russell and Fish, 2016). Race-based unfair treatment, by contrast, often unfolds as repeated and publicly legible devaluation, which may be more likely to elicit anger, vigilance, or oppositional coping (Liu and Li, 2024; Martin-Storey and Benner, 2019). Disability-based unfair treatment may span both pathways because it can combine chronic exclusion with frustration, dependency-related strain, and repeated peer invalidation (Abregú-Crespo et al., 2024; Li et al., 2025b).

These interpretations, however, remain provisional. The observed differences were differences in relative pattern and strength, not evidence that each form of unfair treatment maps onto a wholly discrete mechanism. Moreover, these categories are unlikely to be socially independent. Some youth likely experienced overlapping forms of marginalization, so the coefficients should not be interpreted as pure estimates of isolated identities but as associations tied to analytically separated forms of unfair treatment within potentially intersecting lives (Benner et al., 2018; Pascoe and Smart Richman, 2009; Priest et al., 2013). This caution was also consistent with our exploratory overlap analyses, which suggested the highest predicted burden under triple exposure. Between-category comparisons should therefore be evaluated in terms of substantive magnitude as well as statistical significance, particularly because estimates based on smaller exposure groups may be less precise. The present findings thus support a graded interpretation: all three forms of discrimination were harmful, but their links to internalizing and externalizing difficulties were not interchangeable.

Within those limits, the pattern both validates and refines prior scholarship. Consistent with meta-analytic evidence, all three forms of mistreatment were associated with poorer mental health and greater peer difficulties (Fish et al., 2023; Russell and Fish, 2016). At the same time, our coefficient comparisons suggest that sexual orientation or gender identity-based bias may be more strongly associated with internalizing-linked victimization than racial- or disability-based bias in this sample, whereas race-based unfair treatment may be more closely associated with externalizing responses (Goldbach and Gibbs, 2017; Meyer, 2003). The residual direct association between SOGI-related unfair treatment and perpetration may also reflect unmeasured contextual conditions, such as hostile school climates or peer-network dynamics, as well as measurement constraints in caregiver-reported SOGI exposure. Disability-based unfair treatment showed a less singular profile, with associations spanning both internalizing and externalizing domains, which may reflect the heterogeneous social and developmental challenges faced by youth with disabilities in peer settings (Augustine et al., 2024).

Crucially, the analytical approach we employed extends a literature that has often examined single forms of discrimination in isolation or grouped diverse biases together (Bowleg, 2012; Cole, 2009; Mulvey et al., 2018; Sánchez-Sánchez et al., 2024). By formally contrasting coefficients, we provide comparative evidence that different forms of unfair treatment are not interchangeable in their associations with youth adjustment, even though the categories themselves may overlap and the precision of estimates may vary across subgroups (MacKinnon, 2012; Paternoster et al., 1998). This contribution is best understood not as establishing a simple hierarchy of harm, but as showing that identity-based stress may be patterned differently across social locations. Future research should test whether these contrasts persist after more explicit modeling of intersecting identities, subgroup precision, and contextual moderators.

### Theoretical and practical implications

The present findings carry implications for both theory and practice, although these implications are more qualified than purely confirmatory. At a broad level, the results are consistent with minority stress and developmental psychopathology frameworks in showing that stigmatizing victimization is associated with maladjustment among marginalized youth (Li et al., 2025a). Yet the pattern does not map neatly onto existing theory. Minority stress theory has been especially influential in explaining how prejudice-related experiences become internalized as distress, shame, and mental health disparities (Meyer, 2003; Russell and Fish, 2016). Our results are consistent with that account for sexual minority youth, whose orientation-based victimization was more strongly associated with internalizing difficulties (Gower et al., 2018a; Schrager et al., 2022). At the same time, the fact that racial discrimination was more associated with outward aggression suggests an expansion of theory to accommodate externalizing pathways (Benner et al., 2018; Compas et al., 2017). Additionally, this insight bridges minority stress with developmental theories of stress and coping, indicating that some youth, particularly those facing racism or ableism, may externalize their pain as a protective or reactive strategy (Augustine et al., 2024).

Practically, the findings suggest several directions for prevention and support, but they should not be interpreted as direct evidence for the effectiveness of any particular intervention. Because the analyses are observational and cross-sectional, the study cannot determine whether changes in school policies, counseling practices, or peer programs would alter the outcomes documented here. The results nonetheless indicate that universal anti-bullying efforts may be insufficient when they do not attend to the identity-specific meanings of harassment. This is not because generalized programs lack value, but because they may miss forms of risk that are bound up with stigma, exclusion, and social devaluation.

Within that more cautious frame, our findings are consistent with the potential value of tailored supports. For sexual minority youth, the strong association between orientation-based victimization and internalizing difficulties is consistent with affirming mental health support, trusted adult relationships, and school climates that explicitly reject homophobic harassment (Galán et al., 2021; Gower et al., 2018b). For youth exposed to race- or disability-based victimization, the observed pattern suggests that schools and clinicians may need to assess not only distress but also anger, interpersonal conflict, and other outwardly directed responses, while avoiding the pathologization of reactions that emerge in discriminatory environments (Baams and Russell, 2020; Day et al., 2020; Hatzenbuehler and Keyes, 2013; Marx and Kettrey, 2016).

Across contexts, the present findings suggest that educators and mental health professionals may benefit from training to recognize bias-based harassment as a potentially serious source of harm rather than dismissing it as harmless “kids being kids”. Our findings are also consistent with the potential value of creating safe, affirming environments for all young people, where diversity is celebrated and support is attentive to the unique challenges faced by marginalized youth. Although the present observational design does not permit causal claims about intervention effectiveness, these results point to plausible directions for prevention and support that may help reduce bullying involvement and promote healing and empowerment among vulnerable youth.

### Limitations

While our study makes several contributions, the findings should be interpreted with important limitations in mind. First, the data are cross-sectional and the temporal structure of the measures is not fully aligned: unfair treatment and diagnosis items refer to whether the child had EVER experienced them, whereas bullying victimization and bullying perpetration refer to the past 12 months. Accordingly, the indirect statistical associations reported here should not be interpreted as definitive causal mediation, and reciprocal or reverse-order associations remain possible. Transactional and developmental-cascade perspectives treat maladjustment as the product of ongoing exchanges between youth and their social environments, and longitudinal research indicates bidirectional links between peer victimization and both internalizing and externalizing problems. Stress-generation accounts further suggest that internalizing symptoms may contribute to later interpersonal stress, whereas social-information-processing models imply that aggressive youth may interpret ambiguous cues as hostile or unfair. Our supplementary Bayesian directionality analyses provided stronger support for the mediator-to-outcome ordering than for its reverse in the pairwise comparisons, but support for the unfair-treatment-to-outcome ordering was mixed across domains and broader edge-summary analyses still indicated orientation uncertainty. We therefore interpret the present indirect effects as directionally plausible association patterns rather than definitive evidence of causal mediation.

Second, all focal variables were caregiver-reported. For experiences such as discrimination and bullying, no single report source can be treated as a fully objective or exhaustive benchmark. Adolescent self-reports may be influenced by recall, interpretation, current affect, and willingness to disclose, whereas caregiver proxy reports may miss experiences that were not disclosed or were not visible to adults. Administrative or case-file sources may likewise undercapture experiences that never come to formal attention. This concern is especially relevant for the SOGI item, because some adolescents may not have disclosed their sexual orientation or gender identity to caregivers, while others may have done so; the measure therefore likely misses some experiences and captures others imperfectly. This concern is substantive rather than merely procedural. Parent-proxy and teen self-reports diverge for victimization-related adversities, with greater discordance among LGB+ youth (Licitis et al., 2024). In a national sample of sexual and gender minority youth, roughly one quarter had not disclosed their identities to parents (Pollitt et al., 2023). Sexual orientation- or gender identity-related unfair treatment may therefore be underdetected in the present data, likely rendering estimates for this exposure conservative.

Third, internalizing and externalizing problems were measured with brief diagnosis-based composites, which serve only as proxies for broader psychological functioning.

Finally, the model is intentionally simplified. It isolates a theoretically central pair of psychological correlates, but does not include other potentially relevant mechanisms and contextual factors, such as social support, school climate, coping, identity processes, or intersectional experiences of multiple forms of unfair treatment. A fuller social-ecological specification would require a substantially larger parameter space, and likely higher-order dependencies, than the present linear multiple-mediator framework could estimate without sacrificing interpretability. The highest predicted burden on both bullying outcomes occurred under triple exposure. However, these exploratory analyses do not replace a fully intersectional design with adequate subgroup power.

## Conclusion

This study moves debate from whether bias-based bullying harms youth to how distinct prejudices shape who is hurt and who hurts others. Using a nationally representative sample of U.S. adolescents, this study suggests that different unfair-treatment indicators are associated with bullying victimization and bullying perpetration in partly different patterns. In particular, SOGI-related unfair treatment showed the strongest associations with both outcomes, whereas race- and disability-related unfair treatment were more consistently linked to victimization. Internalizing problems were associated with both bullying outcomes, and externalizing problems appeared particularly relevant in the SOGI-related models. These differences refine minority stress and developmental psychopathology accounts and justify precision prevention: universal protections are necessary, but enumerated, sexuality-specific policies, trustworthy reporting, and affirming school climates are decisive levers, while culturally responsive and disability-inclusive supports should target the internalizing burden that channels youth toward being bullied. Limitations include cross-sectional design, brief measures, and under-representation of sexual minority youth, which counsel caution and motivate longitudinal, intersectional, and quasi-experimental research that follows bully-victims and exploits policy changes. The practical message is clear: naming and addressing specific prejudices, rather than treating bullying as generic misbehavior, is the most credible path to reducing both psychological harm and peer violence.

## Data Availability

This study used publicly available data from the National Survey of Children's Health (NSCH). The de-identified datasets analyzed in this article can be accessed free of charge from the Data Resource Center for Child and Adolescent Health. Further inquiries can be directed to the corresponding author.
