# Supplementary material for: Different stigmas, different patterns: unfair treatment maps onto bullying involvement through internalizing and externalizing problems
Source: Front Psychol. 2026 Jun 18;17:1780810. doi: 10.3389/fpsyg.2026.1780810 (PMC13323299; doi:10.3389/fpsyg.2026.1780810)
Supplement: Supplementary file 1 [file Supplementary_File_1.docx]

**Supplementary Material for:**

**Different stigmas, different patterns: Unfair treatment maps onto bullying involvement through internalizing and externalizing problems**

**Supplementary Note 1. Expanded rationale for modeling bullying-frequency outcomes as ordered scores.**

Although bullying victimization and bullying perpetration were measured as 5-category ordinal frequency variables, they were modeled in the primary mediation analyses as ordered frequency scores. This specification was chosen for three reasons. First, prior bullying research has analyzed comparable frequency measures as continuous, including mediation settings and analyses based on mean item scores (van Hoof et al., 2008; Kljakovic et al., 2015). Second, methodological work suggests that treating 5-category ordinal outcomes as approximately continuous is often acceptable in large samples and generally entails limited distortion of substantive conclusions (Norman, 2010; Rhemtulla et al., 2012). Third, the linear specification allowed all paths in the mediation models to be estimated on a common 0-1 percentage scale, thereby facilitating *b_p_* estimation and coefficient comparison across equations (Jiang et al., 2021; Zhao et al., 2024; Zhao et al., 2025). This logic is also consistent with earlier applied work using bounded 0-1 scaling to improve interpretability and cross-model comparison (Luo & Homburg, 2008). Conceptually, the response options capture a graded increase in bullying recurrence or chronicity, although they do not constitute a perfectly interval-scaled metric. Ordered logistic regression is a reasonable alternative, but it imposes the proportional-odds assumption and yields cumulative log-odds parameters that are less directly comparable across mediation paths (Williams, 2016). Accordingly, the coefficients are interpreted as associations with higher levels of bullying frequency rather than as exact unit changes on a strictly interval scale.

**Supplementary Note 2. Expanded interpretation of the survey-weighted robustness analyses.**

Because the NSCH is based on a complex survey design and provides sampling weights, we conducted additional robustness analyses to evaluate whether the main conclusions were sensitive to survey-weight adjustment. PROCESS v5.3 does not directly accommodate NSCH sampling weights or design-based variance estimation within the mediation framework used in the primary analyses. Therefore, the PROCESS estimates should be interpreted as unweighted association models. To address this limitation, we re-estimated the mediation models in R using the lavaan package, incorporating the NSCH sampling weights and Huber-White heteroskedasticity-robust standard errors (Huber, 1967; White, 1980; Rosseel, 2012; Savalei & Rosseel, 2022). These weighted robustness analyses largely reproduced the primary pattern of findings. The three unfair-treatment indicators remained positively associated with bullying victimization, SOGI-related unfair treatment remained the strongest and most consistent correlate across models, and the internalizing-related indirect associations remained statistically detectable for all three unfair-treatment indicators in both outcomes. At the same time, some relative magnitudes varied under weighting, most notably in the bullying-perpetration model, where a small positive direct association with race-based unfair treatment emerged and externalizing problems exceeded internalizing problems in magnitude. We therefore treat the weighted models as a robustness check that broadly supports, but does not identically replicate, the primary unweighted association models. Full weighted results are reported in the Supplementary Material (see Supplementary Tables 2-5).

**Supplementary Table 1. Survey-weighted descriptive characteristics of the analytic samples.**

| **Characteristic** | **Model 1** | **Model 2** |
| --- | --- | --- |
| N | 30400 | 30389 |
| Unfair treatment by race | 1440 (5.17%) | 1440 (5.17%) |
| Unfair treatment by sexual orientation or gender identity | 1334 (3.69%) | 1334 (3.69%) |
| Unfair treatment by disability | 691 (1.96%) | 689 (1.95%) |
| Selected child’ sex |  |  |
| Female | 14591 (48.61%) | 14582 (48.61%) |
| Male | 15809 (51.39%) | 15807 (51.39%) |
| Mom’ age (mean) | 29.44 | 29.44 |
| Selected child’ race |  |  |
| Hispanic | 4537 (26.20%) | 4529 (26.23%) |
| Non-Hispanic Asian | 1773 (4.72%) | 1775 (4.75%) |
| Non-Hispanic Black | 1818 (11.68%) | 1822 (11.69%) |
| Non-Hispanic Multirace | 2328 (7.54%) | 2326 (7.54%) |
| Non-Hispanic White | 19944 (49.85%) | 19937 (49.80%) |
| Caregiver sex |  |  |
| Female | 20891 (68.66%) | 20888 (68.67%) |
| Male | 9509 (31.34%) | 9501 (31.33%) |
| Caregiver’ employment |  |  |
| Employed full-time | 21092 (65.47%) | 21087 (65.45%) |
| Employed part-time | 3699 (12.89%) | 3690 (12.86%) |
| Working WITHOUT pay | 392 (1.38%) | 392 (1.38%) |
| Not employed but looking for work | 1232 (5.44%) | 1230 (5.44%) |
| Not employed and not looking for work | 3985 (14.82%) | 3990 (14.86%) |
| Caregiver’ physical health (mean) | 2.21 | 2.21 |
| Caregiver’ mental health (mean) | 2.10 | 2.10 |
| Household income as a percentage of the Federal Poverty Level |  |  |
| 0-99% FPL | 3548 (16.19%) | 3538 (16.20%) |
| 100-199% FPL | 4894 (19.68%) | 4892 (19.65%) |
| 200-399% FPL | 8995 (29.82%) | 8999 (29.84%) |
| 400% FPL or greater | 12963 (34.30%) | 12960 (34.31%) |
| Number of family members in 2022 |  |  |
| 1 Member | 2332 (5.95%) | 2332 (5.93%) |
| 2 Members | 8515 (19.83%) | 8508 (19.82%) |
| 3 Members | 11558 (33.34%) | 11562 (33.38%) |
| 4 Members | 5409 (26.77%) | 5409 (26.77%) |
| 5 Members | 2586 (14.11%) | 2578 (14.10%) |
| Caregiver’ relationship with selected child |  |  |
| Biological or adoptive parent | 27732 (89.28%) | 27719 (89.28%) |
| Step-parent | 757 (2.66%) | 755 (2.66%) |
| Foster parent | 58 (0.34%) | 58 (0.34%) |
| Grand parent | 1414 (5.69%) | 1416 (5.69%) |
| Other: Non-Relative | 345 (1.65%) | 348 (1.65%) |
| Other: Relative | 94 (0.38%) | 93 (0.38%) |
| Caregiver born in the U.S. |  |  |
| Yes | 25677 (77.80%) | 25671 (77.75%) |
| No | 4723 (22.20%) | 4718 (22.25%) |
| Internalizing problems | 2281 (5.56%) | 2282 (5.58%) |
| Externalizing problems | 2165 (6.29%) | 2164 (6.27%) |
| Bullying victimization (mean) | 1.56 | 1.56 |
| Bullying perpetration (mean) | 1.18 | 1.18 |
|  |  |  |

Note. Values are unweighted n (weighted %) for categorical variables and weighted means for continuous variables. The Model 1 and Model 2 labels are retained for direct comparability with the manuscript.

**Supplementary Table 2. Survey-weighted direct effects in Model 1.**

|  | **Internalizing problems** | **Externalizing problems** | **Bullying victimization** |
| --- | --- | --- | --- |
|  | ***b_p_* (SE)** | ***b_p_* (SE)** | ***b_p_* (SE)** |
| Unfair treatment by race | .030*** (.006) | -.003 (.006) | .072*** (.005) |
| Unfair treatment by sexual orientation or gender identity | .185*** (.007) | .210*** (.007) | .211*** (.006) |
| Unfair treatment by disability | .268*** (.009) | .049*** (.010) | .066*** (.009) |
| Selected child’ sex (1 = Female) | .034*** (.002) | -.054*** (.003) | .014*** (.002) |
| Mom’ age | -.038*** (.006) | -.015* (.007) | .011* (.005) |
| Selected child’ race (reference “Black”) |  |  |  |
| Hispanic | .018*** (.005) | .003 (.005) | .012** (.004) |
| White | .033*** (.004) | .010* (.005) | .045*** (.004) |
| Asian | .008 (.007) | -.001 (.008) | .002 (.007) |
| Multiracial | .027*** (.006) | .014* (.006) | .018*** (.005) |
| Caregiver sex (1 = Female) | .016*** (.003) | .007* (.003) | .022*** (.003) |
| Caregiver’ employment (reference “Not employed and not looking for work”) |  |  |  |
| Employed full-time | -.002 (.004) | -.018*** (.004) | .021*** (.003) |
| Employed part-time | -.005 (.005) | -.016** (.005) | .015*** (.004) |
| Employed without pay | .018 (.011) | -.021 (.012) | .041*** (.010) |
| Not employed but looking for work | .014* (.006) | -.015* (.007) | .031*** (.006) |
| Caregiver’ physical health | -.032*** (.007) | -.041*** (.008) | -.056*** (.006) |
| Caregiver’ mental health | -.076*** (.007) | -.049*** (.007) | -.110*** (.006) |
| Household income as a percentage of the federal poverty level | -.003 (.004) | -.030*** (.004) | .020*** (.004) |
| Number of family members in 2022 | -.040*** (.005) | -.046*** (.005) | .007 (.004) |
| Caregiver’ relationship with selected child (reference “Other: Relative”) |  |  |  |
| Biological or adoptive parent | .000 (.020) | -.004 (.022) | -.002 (.018) |
| Step-parent | .046* (.022) | .045 (.023) | .002 (.020) |
| Foster parent | .202*** (.030) | .138*** (.032) | -.006 (.027) |
| Grand parent | .012 (.021) | .023 (.023) | .018 (.019) |
| Other: Non-Relative | .041 (.023) | .048* (.024) | -.033 (.020) |
| Caregiver born in the U.S. (1 = outside the US) | -.014*** (.004) | -.021*** (.004) | -.031*** (.003) |
| Internalizing problems |  |  | .092*** (.005) |
| Externalizing problems |  |  | .146*** (.005) |
| N | 30389 | 30389 | 30389 |
| R-squared | .107 | .070 | .190 |
| Adj R-squared | .107 | .069 | .189 |

Note. Entries are range-standardized coefficients with weighted standard errors in parentheses from survey-weighted linear models. The outward naming convention and the Model 1/Model 2 labels are retained for direct comparability with the manuscript.

**p* < 0.05, ***p* < 0.01, ****p* < 0.001.

**Supplementary Table 3. Survey-weighted direct effects in Model 2.**

|  | **Internalizing problems** | **Externalizing problems** | **Bullying perpetration** |
| --- | --- | --- | --- |
|  | ***b_p_* (SE)** | ***b_p_* (SE)** | ***b_p_* (SE)** |
| Unfair treatment by race | .030*** (.006) | -.003 (.006) | .019*** (.003) |
| Unfair treatment by sexual orientation or gender identity | .184*** (.007) | .209*** (.007) | .049*** (.004) |
| Unfair treatment by disability | .273*** (.009) | .049*** (.010) | -.008 (.005) |
| Selected child’ sex (1 = Female) | .033*** (.002) | -.055*** (.003) | -.003 (.001) |
| Mom’ age | -.039*** (.006) | -.016* (.007) | -.008* (.003) |
| Selected child’ race (reference “Black”) |  |  |  |
| Hispanic | .018*** (.005) | .003 (.005) | -.002 (.003) |
| White | .033*** (.004) | .011* (.005) | .014*** (.002) |
| Asian | .008 (.007) | -.001 (.008) | -.001 (.004) |
| Multiracial | .027*** (.006) | .014* (.006) | .009** (.003) |
| Caregiver sex (1 = Female) | .017*** (.003) | .007* (.003) | .007*** (.002) |
| Caregiver’ employment (reference “Not employed and not looking for work”) |  |  |  |
| Employed full-time | -.002 (.004) | -.018*** (.004) | .002 (.002) |
| Employed part-time | -.005 (.005) | -.016** (.005) | .002 (.003) |
| Employed without pay | .018 (.011) | -.022 (.012) | -.000 (.006) |
| Not employed but looking for work | .014* (.006) | -.016* (.007) | .004 (.004) |
| Caregiver’ physical health | -.032*** (.007) | -.041*** (.008) | .002 (.004) |
| Caregiver’ mental health | -.075*** (.007) | -.050*** (.007) | -.051*** (.004) |
| Household income as a percentage of the federal poverty level | -.004 (.004) | -.031*** (.004) | .006* (.002) |
| Number of family members in 2022 | -.041*** (.005) | -.046*** (.005) | -.006* (.003) |
| Caregiver’ relationship with selected child (reference “Other: Relative”) |  |  |  |
| Biological or adoptive parent | .001 (.020) | -.003 (.022) | -.001 (.012) |
| Step-parent | .046* (.022) | .045 (.023) | .064*** (.012) |
| Foster parent | .203*** (.030) | .139*** (.032) | .066*** (.017) |
| Grand parent | .013 (.021) | .024 (.023) | -.001 (.012) |
| Other: Non-Relative | .042 (.022) | .048* (.024) | .014 (.013) |
| Caregiver born in the U.S. (1 = outside the US) | -.013*** (.004) | -.021*** (.004) | -.018*** (.002) |
| Internalizing problems |  |  | .034*** (.003) |
| Externalizing problems |  |  | .114*** (.003) |
| N | 30400 | 30400 | 30400 |
| R-squared | .109 | .070 | .112 |
| Adj R-squared | .108 | .070 | .112 |

Note. Entries are range-standardized coefficients with weighted standard errors in parentheses from survey-weighted linear models. The outward naming convention and the Model 1/Model 2 labels are retained for direct comparability with the manuscript.

**p* < 0.05, ***p* < 0.01, ****p* < 0.001.

**Supplementary Table 4. Survey-weighted indirect effects in Model 1.**

| **Indirect path** | ***b_p_* (SE)** | **95% CI** | ***p*** |
| --- | --- | --- | --- |
| UTR→IP→BV | .003* (.001) | .000, .006 | 0.024 |
| UTSO→IP→BV | .017*** (.003) | .012, .024 | 0.000 |
| UTD→IP→BV | .025*** (.004) | .018, .032 | 0.000 |
| UTR→EP→BV | -.000 (.002) | -.004, .003 | 0.792 |
| UTSO→EP→BV | .031*** (.004) | .023, .040 | 0.000 |
| UTD→EP→BV | .007 (.005) | -.001, .017 | 0.088 |

Note. Entries are range-standardized indirect effects with bootstrap standard errors in parentheses. Confidence intervals are percentile bootstrap intervals obtained from survey-weighted cluster resampling. UTR, unfair treatment by race; UTSO, unfair treatment by sexual orientation or gender identity; UTD, unfair treatment by disability; IP, internalizing problems; EP, externalizing problems; BV, bullying victimization.

**p* < 0.05, ***p* < 0.01, ****p* < 0.001.

**Supplementary Table 5. Survey-weighted indirect effects in Model 2.**

| **Indirect path** | ***b_p_* (SE)** | **95% CI** | ***p*** |
| --- | --- | --- | --- |
| UTR→IP→BP | .001* (.001) | .000, .002 | 0.028 |
| UTSO→IP→BP | .006*** (.002) | .003, .010 | 0.000 |
| UTD→IP→BP | .009*** (.002) | .005, .015 | 0.000 |
| UTR→EP→BP | -.000 (.001) | -.003, .002 | 0.848 |
| UTSO→EP→BP | .024*** (.004) | .018, .032 | 0.000 |
| UTD→EP→BP | .006 (.004) | -.001, .013 | 0.140 |

Note. Entries are range-standardized indirect effects with bootstrap standard errors in parentheses. Confidence intervals are percentile bootstrap intervals obtained from survey-weighted cluster resampling. UTR, unfair treatment by race; UTSO, unfair treatment by sexual orientation or gender identity; UTD, unfair treatment by disability; IP, internalizing problems; EP, externalizing problems; BP, bullying perpetration.

**p* < 0.05, ***p* < 0.01, ****p* < 0.001.

**Supplementary Note 3. Interpreting the Bayesian directional-plausibility analyses.**

This note provides the fuller interpretation of the Bayesian directional-plausibility analyses. As an additional sensitivity analysis motivated by concerns about reciprocal ordering, we estimated Bayesian directional-plausibility models comparing the hypothesized ordering of unfair treatment, psychological ill-being, and bullying involvement with selected reverse-order alternatives. Given the cross-sectional design, these models were used to rank the relative compatibility of candidate directions rather than to establish causality. The pairwise comparisons provided stronger support for the ordering of internalizing and externalizing problems prior to both bullying victimization and bullying perpetration than for the reverse ordering. By contrast, support for the unfair-treatment-to-outcome ordering was mixed across exposure domains, and the broader edge-summary analyses still indicated directional uncertainty in the focal-node graph. These results therefore provide graded directional plausibility rather than definitive evidence of temporal sequence or causal mediation.

**Supplementary Note 4. Expanded narrative summary of the coefficient-comparison matrices (Tables 6-9).**

To keep the main Results section readable, the more detailed narrative interpretation of the main-text coefficient-comparison matrices is provided here. Survey-weighted scalar-comparison matrices are reported separately in Supplementary Tables 6-9. The *d_s_* matrices are intended as comparative tools rather than stand-alone effect estimates; they help identify which association patterns were relatively larger on a common *b_p_* scale. In Table 6, for direct associations of Model 1, the SOGI-related unfair treatment coefficient for bullying victimization was the largest among the three unfair-treatment indicators, exceeding race and disability (race: *d_s_* = .133***; disability: *d_s_* = .144***); the disability-race contrast was statistically inconclusive (*d_s_* = .011, not significant). At the endpoint, externalizing exceeded internalizing (*d_s_* = .041***). Upstream comparisons located mechanisms: disability-internalizing exceeded SOGI-internalizing (*d_s_* = .091***), whereas SOGI-externalizing was greater than race and disability (race: *d_s_* = .255***; disability: *d_s_* = .259***). For indirect associations, in Table 7, the most efficient route to victimization was SOGI-related unfairness via externalizing, larger than all alternatives (race via internalizing: *d_s_* = .036***; disability via internalizing: *d_s_* = .011**; race via externalizing: *d_s_* = .037***; disability via externalizing: *d_s_* = .038***; SOGI via internalizing: *d_s_* = .020***). Within internalizing-associated routes, disability via internalizing exceeded both race and SOGI (vs race: *d_s_* = .025***; vs SOGI: *d_s_* = .010***). Collectively, these contrasts strengthened H1b over H1a/H1c, substantiated H2 for victimization, and specified H3 by showing SOGI-based stigma transmitting risk chiefly through externalizing, whereas ableism concentrated through internalizing.

In Model 2, for direct associations in Table 8, the SOGI-related unfairness-perpetration association surpassed race and disability (race: *d_s_* = .041***; disability: *d_s_* = .051***). Here the internalizing-perpetration coefficient was larger than the externalizing-perpetration coefficient (*d_s_* = .088***). Upstream, SOGI-externalizing again dominated race and disability (race: *d_s_* = .254***; disability: *d_s_* = .258***). For indirect associations, in Table 9, the SOGI-related unfairness via externalizing was uniquely largest (vs race via internalizing: *d_s_* = .031***; vs disability via internalizing: *d_s_* = .023***; vs SOGI via internalizing: *d_s_* = .026***; vs race via externalizing: *d_s_* = .030***; vs disability via externalizing: *d_s_* = .030***). Internalizing-associated effects were smaller but nonzero; disability via internalizing slightly exceeded race and SOGI (vs race: *d_s_* = .007***; vs SOGI: *d_s_* = .003***). The *d_s_* matrices therefore most strongly corroborated H1b and H3, provided qualified support for H1a/H1c, and further affirmed H2 across roles. Accordingly, these matrices are best read as comparative ranking devices that help interpret relative emphasis across pathways, not as independent evidence of mechanistic ordering.

**Supplementary Figure 1. Weighted SHAP importance profile for bullying victimization.**


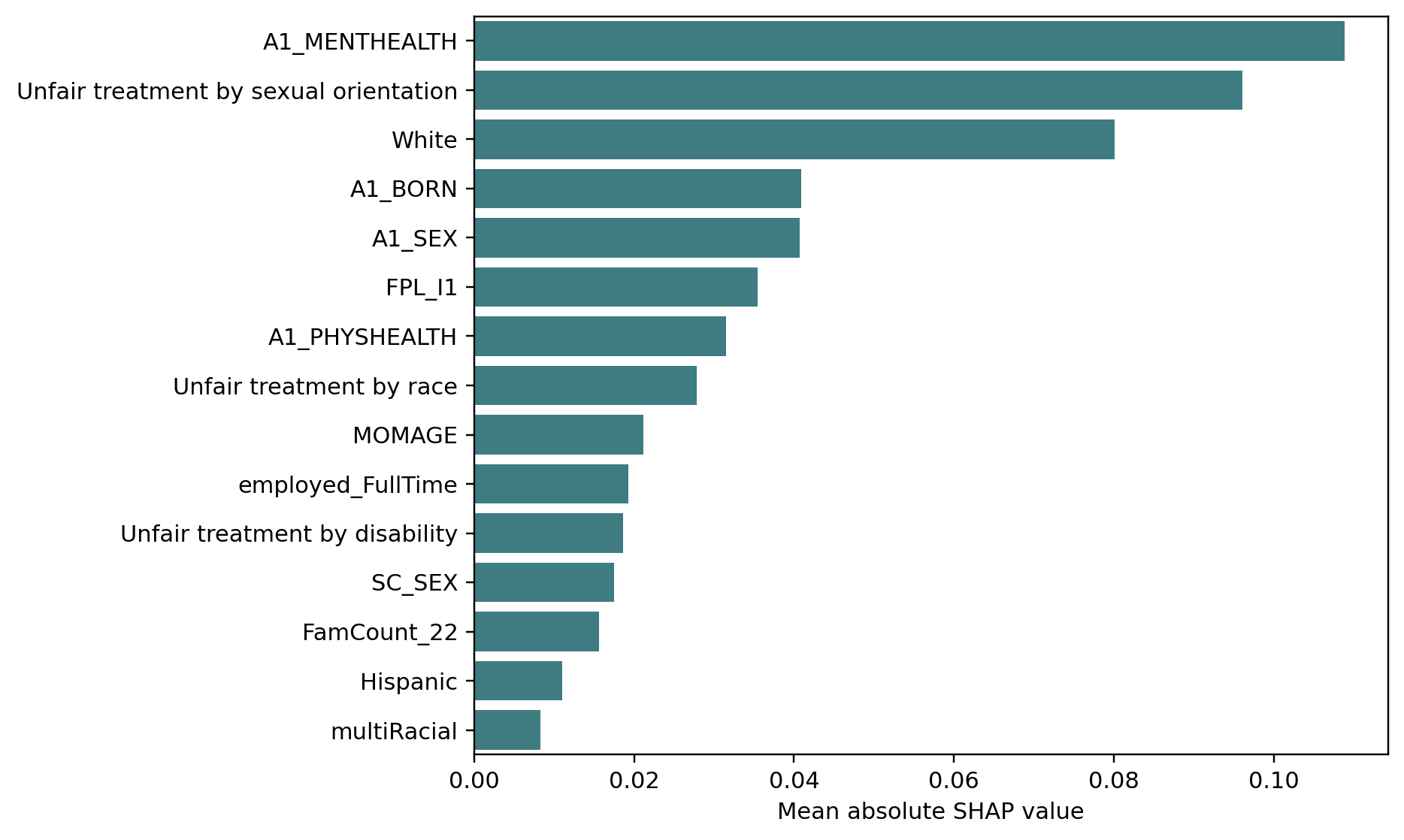


Note. Larger values indicate greater average contribution to the fitted weighted prediction model.

**Supplementary Table 6. Survey-weighted scalar comparison of efficiencies for direct paths in Model 1.**

| **path(i) path(j)** | **UTR→IP** | **UTSO→IP** | **UTD→IP** | **UTR→EP** | **UTSO→EP** | **UTD→EP** | **UTR→BV** | **UTSO→BV** | **UTD→BV** | **EP→BV** | **IP→BV** |
| --- | --- | --- | --- | --- | --- | --- | --- | --- | --- | --- | --- |
| UTR→IP | — | .155*** | .238*** | -.027 | .180*** | .019 | .042* | .181*** | .036 | .116*** | .062*** |
| UTSO→IP | -.155*** | — | .084* | -.182*** | .025 | -.136*** | -.113*** | .027 | -.119*** | -.039 | -.093*** |
| UTD→IP | -.238*** | -.084* | — | -.265*** | -.059 | -.220*** | -.197*** | -.057 | -.203*** | -.122*** | -.176*** |
| UTR→EP | .027 | .182*** | .265*** | — | .207*** | .046 | .069*** | .208*** | .063** | .143*** | .089*** |
| UTSO→EP | -.180*** | -.025 | .059 | -.207*** | — | -.161*** | -.138*** | .002 | -.144*** | -.064* | -.118*** |
| UTD→EP | -.019 | .136*** | .220*** | -.046 | .161*** | — | .023 | .162*** | .017 | .097* | .043 |
| UTR→BV | -.042* | .113*** | .197*** | -.069*** | .138*** | -.023 | — | .139*** | -.006 | .074*** | .020 |
| UTSO→BV | -.181*** | -.027 | .057 | -.208*** | -.002 | -.162*** | -.139*** | — | -.145*** | -.065** | -.119*** |
| UTD→BV | -.036 | .119*** | .203*** | -.063** | .144*** | -.017 | .006 | .145*** | — | .080*** | .026 |
| EP→BV | -.116*** | .039 | .122*** | -.143*** | .064* | -.097* | -.074*** | .065** | -.080*** | — | -.054** |
| IP→BV | -.062*** | .093*** | .176*** | -.089*** | .118*** | -.043 | -.020 | .119*** | -.026 | .054** | — |

Note. Main cells show scalar differences between efficiencies (*d_s_* = |*b_p_*(i)| - |*b_p_*(j)|). Diagonal entries are em dashes. UTR, unfair treatment by race; UTSO, unfair treatment by sexual orientation or gender identity; UTD, unfair treatment by disability; IP, internalizing problems; EP, externalizing problems; BV, bullying victimization.

**p* < 0.05, ***p* < 0.01, ****p* < 0.001.

**Supplementary Figure 2. Weighted SHAP importance profile for bullying perpetration.**


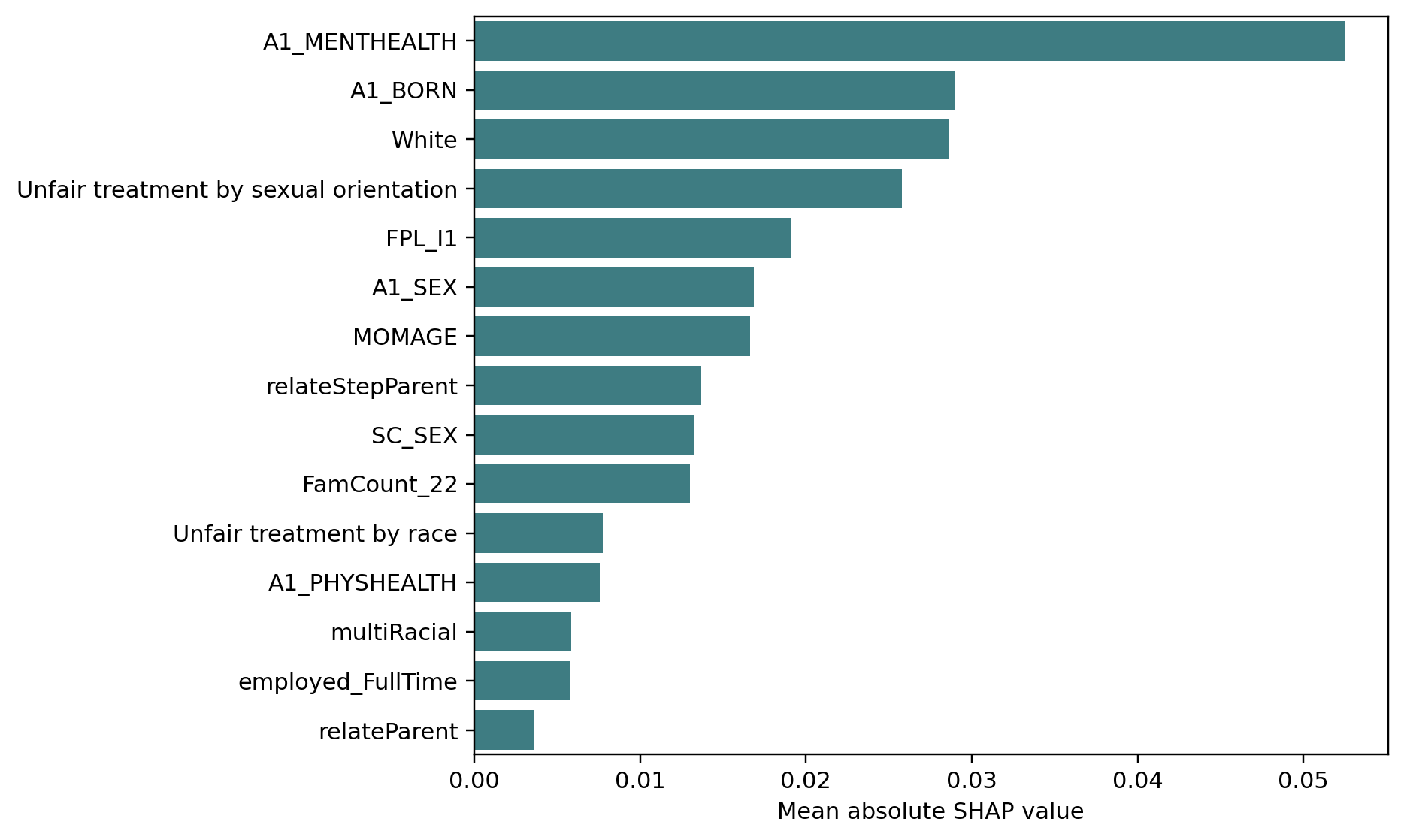


Note. Larger values indicate greater average contribution to the fitted weighted prediction model.

**Supplementary Table 7. Survey-weighted scalar comparison of efficiencies for indirect paths in Model 1.**

| **path(i) path(j)** | **UTR→IP→BV** | **UTSO→IP→BV** | **UTD→IP→BV** | **UTR→EP→BV** | **UTSO→EP→BV** | **UTD→EP→BV** |
| --- | --- | --- | --- | --- | --- | --- |
| UTR→IP→BV | — | .014*** | .022*** | -.002 | .028*** | .004 |
| UTSO→IP→BV | -.014*** | — | .008* | -.017*** | .014* | -.010 |
| UTD→IP→BV | -.022*** | -.008* | — | -.024*** | .006 | -.018* |
| UTR→EP→BV | .002 | .017*** | .024*** | — | .030*** | .007 |
| UTSO→EP→BV | -.028*** | -.014* | -.006 | -.030*** | — | -.024*** |
| UTD→EP→BV | -.004 | .010 | .018* | -.007 | .024*** | — |

Note. Main cells show scalar differences between efficiencies (*d_s_* = |*b_p_*(i)| - |*b_p_*(j)|). Diagonal entries are em dashes. UTR, unfair treatment by race; UTSO, unfair treatment by sexual orientation or gender identity; UTD, unfair treatment by disability; IP, internalizing problems; EP, externalizing problems; BV, bullying victimization.

**p* < 0.05, ***p* < 0.01, ****p* < 0.001.

**Supplementary Figure 3. Pairwise SHAP interaction structure across the three unfair-treatment domains.**


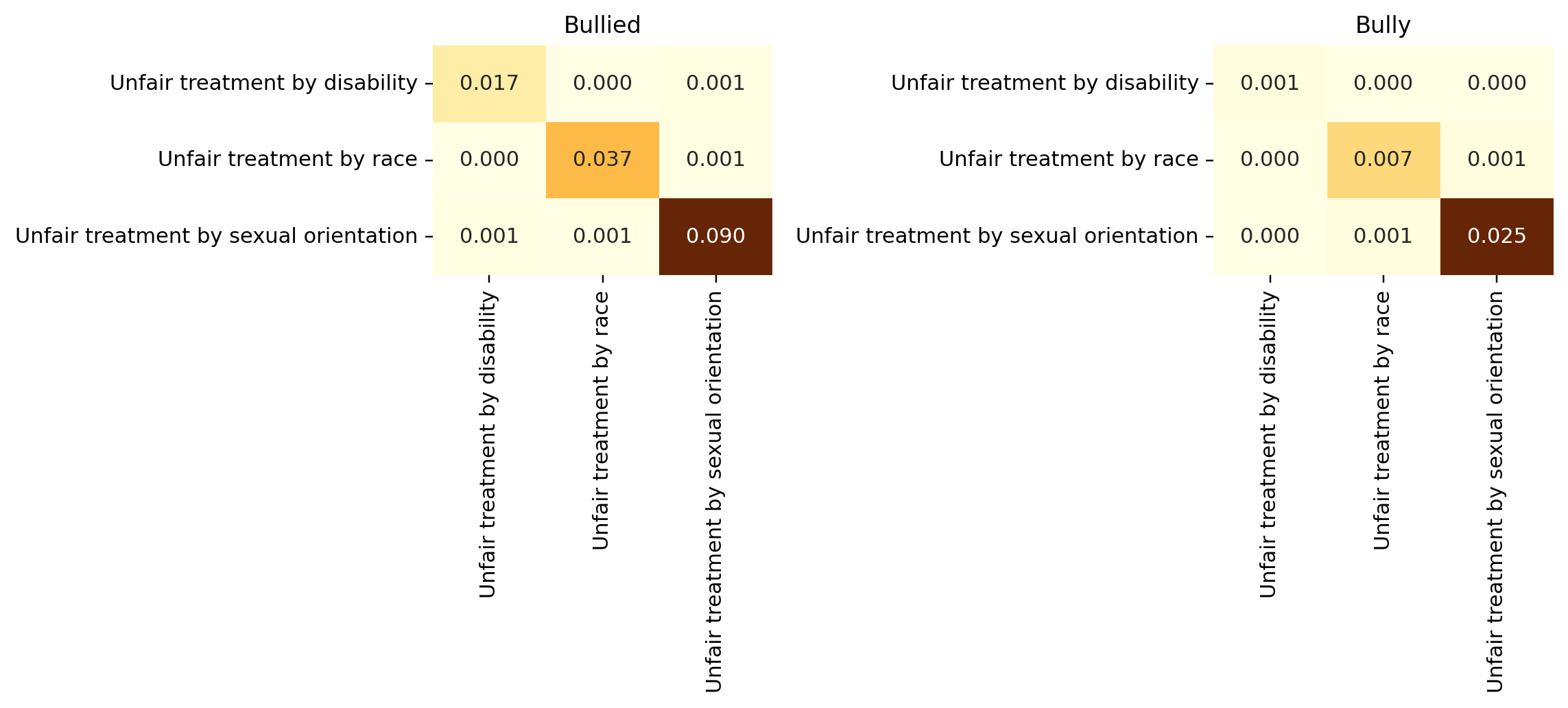


Note. Warmer cells indicate more pronounced pairwise interaction structure in the weighted prediction models.

**Supplementary Table 8. Survey-weighted scalar comparison of efficiencies for direct paths in Model 2.**

| **path(i) path(j)** | **UTR→IP** | **UTD→IP** | **UTSO→IP** | **UTR→EP** | **UTD→EP** | **UTSO→EP** | **UTR→BP** | **UTD→BP** | **UTSO→BP** | **EP→BP** | **IP→BP** |
| --- | --- | --- | --- | --- | --- | --- | --- | --- | --- | --- | --- |
| UTR→IP | — | .243*** | .154*** | -.027 | .019 | .179*** | -.011 | -.022 | .019 | .084*** | .004 |
| UTD→IP | -.243*** | — | -.088* | -.270*** | -.224*** | -.064 | -.253*** | -.265*** | -.223*** | -.159*** | -.239*** |
| UTSO→IP | -.154*** | .088* | — | -.181*** | -.136** | .025 | -.165*** | -.176*** | -.135*** | -.071** | -.151*** |
| UTR→EP | .027 | .270*** | .181*** | — | .045 | .206*** | .016 | .005 | .046** | .111*** | .031* |
| UTD→EP | -.019 | .224*** | .136** | -.045 | — | .161*** | -.029 | -.040 | .001 | .065 | -.015 |
| UTSO→EP | -.179*** | .064 | -.025 | -.206*** | -.161*** | — | -.190*** | -.201*** | -.160*** | -.096*** | -.175*** |
| UTR→BP | .011 | .253*** | .165*** | -.016 | .029 | .190*** | — | -.011 | .030* | .094*** | .014 |
| UTD→BP | .022 | .265*** | .176*** | -.005 | .040 | .201*** | .011 | — | .041** | .106*** | .026* |
| UTSO→BP | -.019 | .223*** | .135*** | -.046** | -.001 | .160*** | -.030* | -.041** | — | .064*** | -.015 |
| EP→BP | -.084*** | .159*** | .071** | -.111*** | -.065 | .096*** | -.094*** | -.106*** | -.064*** | — | -.080*** |
| IP→BP | -.004 | .239*** | .151*** | -.031* | .015 | .175*** | -.014 | -.026* | .015 | .080*** | — |

Note. Main cells show scalar differences between efficiencies (*d_s_* = |*b_p_*(i)| - |*b_p_*(j)|). Diagonal entries are em dashes. UTR, unfair treatment by race; UTSO, unfair treatment by sexual orientation or gender identity; UTD, unfair treatment by disability; IP, internalizing problems; EP, externalizing problems; BP, bullying perpetration.

**p* < 0.05, ***p* < 0.01, ****p* < 0.001.

**Supplementary Figure 4. Weighted predicted means across all eight exposure combinations.**


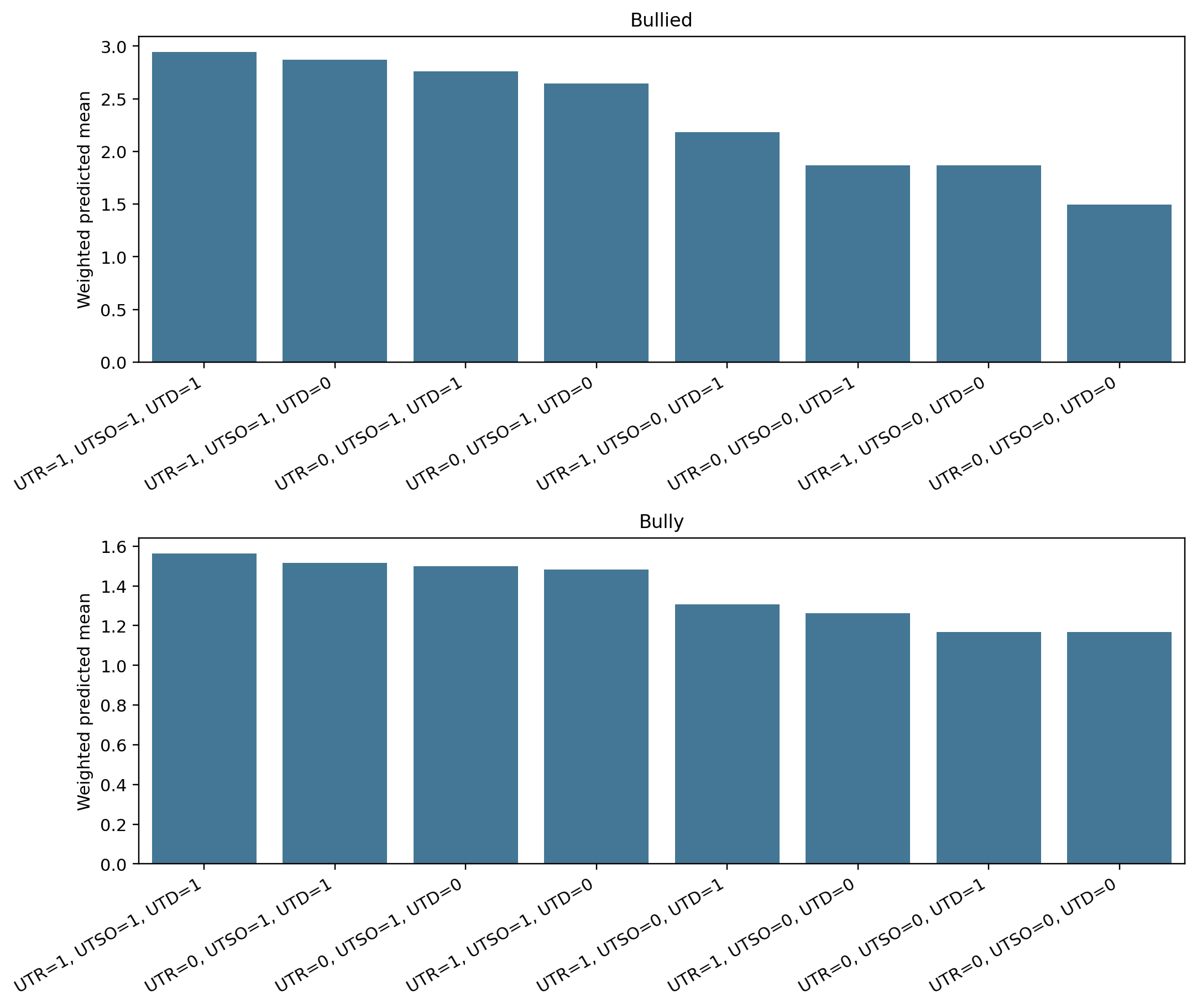


Note. Bars are ordered from the highest to the lowest weighted predicted mean within each outcome.

**Supplementary Table 9. Survey-weighted scalar comparison of efficiencies for indirect paths in Model 2.**

| **path(i) path(j)** | **UTR→IP→BP** | **UTD→IP→BP** | **UTSO→IP→BP** | **UTR→EP→BP** | **UTD→EP→BP** | **UTSO→EP→BP** |
| --- | --- | --- | --- | --- | --- | --- |
| UTR→IP→BP | — | .008*** | .005*** | -.001 | .005 | .023*** |
| UTD→IP→BP | -.008*** | — | -.003* | -.009*** | -.004 | .015*** |
| UTSO→IP→BP | -.005*** | .003* | — | -.006** | -.001 | .018*** |
| UTR→EP→BP | .001 | .009*** | .006** | — | .005 | .023*** |
| UTD→EP→BP | -.005 | .004 | .001 | -.005 | — | .018*** |
| UTSO→EP→BP | -.023*** | -.015*** | -.018*** | -.023*** | -.018*** | — |

Note. Main cells show scalar differences between efficiencies (*d_s_* = |*b_p_*(i)| - |*b_p_*(j)|). Diagonal entries are em dashes. UTR, unfair treatment by race; UTSO, unfair treatment by sexual orientation or gender identity; UTD, unfair treatment by disability; IP, internalizing problems; EP, externalizing problems; BP, bullying perpetration.

**p* < 0.05, ***p* < 0.01, ****p* < 0.001.

**Supplementary Figure 5. Interpretive summary of cumulative exposure patterns.**


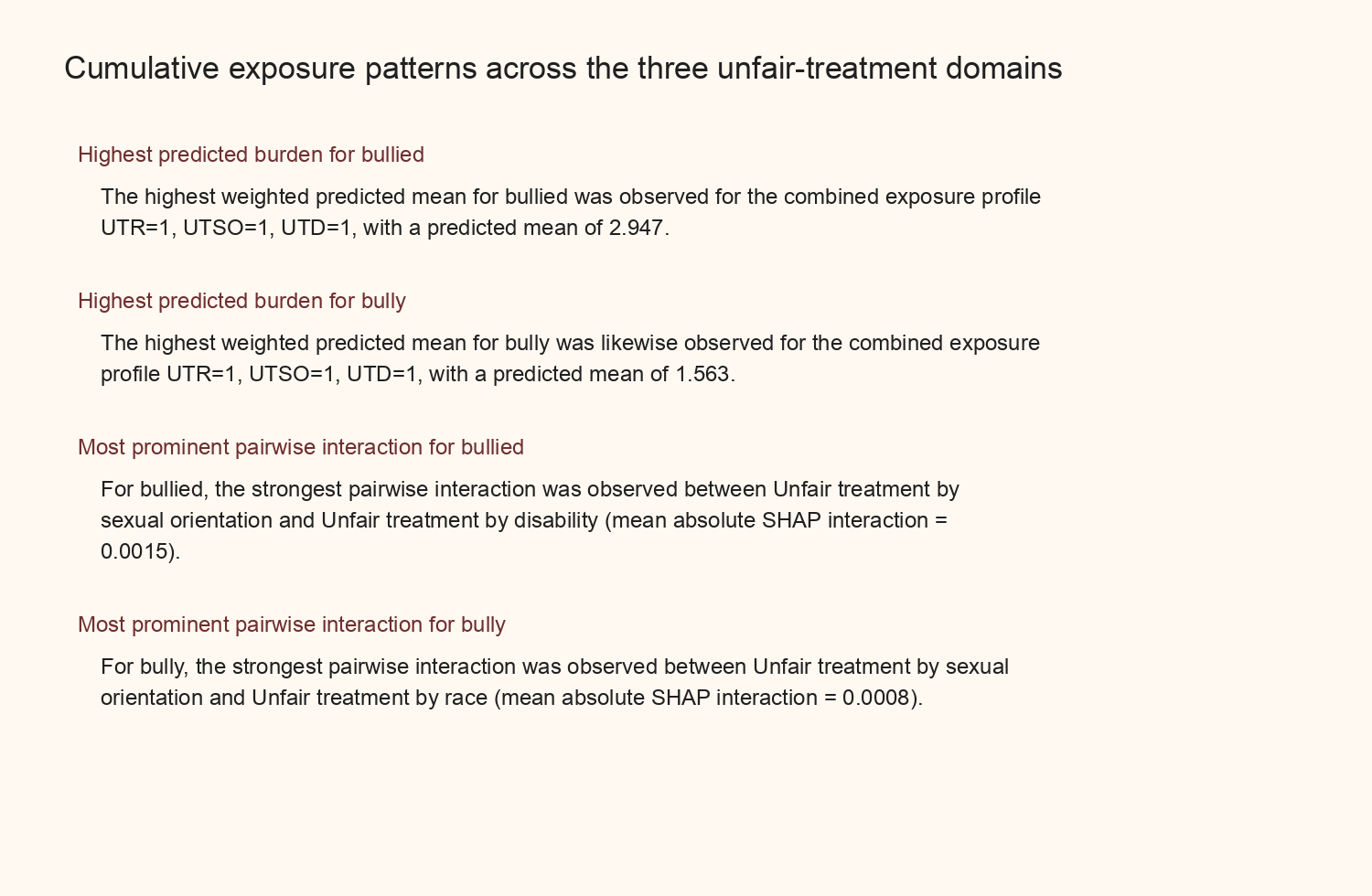


Note. The summary panel translates the weighted machine-learning results into concise substantive statements while retaining the outward naming convention used in the manuscript.

**Supplementary Table 10. Directional support for reviewer-sensitive mediator-outcome orderings.**

| **Outcome** | **Comparison** | **More plausible direction** | **Directional support** |
| --- | --- | --- | --- |
| Bullying victimization | Internalizing problems and Bullying victimization | Internalizing problems→ Bullying victimization | 1.000 |
| Bullying victimization | Externalizing problems and Bullying victimization | Externalizing problems→ Bullying victimization | 1.000 |
| Bullying perpetration | Internalizing problems and Bullying perpetration | Internalizing problems→ Bullying perpetration | 1.000 |
| Bullying perpetration | Externalizing problems and Bullying perpetration | Externalizing problems→ Bullying perpetration | 1.000 |

Note. Directional support values closer to 1 indicate stronger relative compatibility with the reported ordering than with its reverse ordering, conditional on the covariates used in the main analyses. These estimates rank directional plausibility and should not be read as definitive causal proof.

**Supplementary Table 11. Directional support for unfair-treatment exposure and bullied or bully outcomes.**

| **Outcome** | **Comparison** | **More plausible direction** | **Directional support** |
| --- | --- | --- | --- |
| Bullying victimization | Unfair treatment by race and Bullying victimization | Unfair treatment by race→ Bullying victimization | 0.911 |
| Bullying victimization | Unfair treatment by sexual orientation or gender identity and Bullying victimization | Unfair treatment by sexual orientation or gender identity→ Bullying victimization | 1.000 |
| Bullying victimization | Unfair treatment by disability and Bullying victimization | Unfair treatment by disability→ Bullying victimization | 1.000 |
| Bullying perpetration | Unfair treatment by race and Bullying perpetration | Unfair treatment by race→ Bullying perpetration | 0.693 |
| Bullying perpetration | Unfair treatment by sexual orientation or gender identity and Bullying perpetration | Unfair treatment by sexual orientation or gender identity→ Bullying perpetration | 1.000 |
| Bullying perpetration | Unfair treatment by disability and Bullying perpetration | Bullying perpetration→Unfair treatment by disability | 0.745 |

Note. Directional support values closer to 1 indicate stronger relative compatibility with the reported ordering than with its reverse ordering, conditional on the covariates used in the main analyses. These estimates rank directional plausibility and should not be read as definitive causal proof.

**Supplementary Table 12. Edge-presence and edge-direction summary across the focal nodes.**

| **Outcome** | **Node A** | **Node B** | **Edge presence probability** | **More plausible direction** | **Directional support** |
| --- | --- | --- | --- | --- | --- |
| Bullying victimization | Unfair treatment by sexual orientation or gender identity | Bullying victimization | 1.000 | Unfair treatment by sexual orientation or gender identity→ Bullying victimization | 0.670 |
| Bullying victimization | Unfair treatment by disability | Internalizing problems | 1.000 | Unfair treatment by disability→ Internalizing problems | 0.610 |
| Bullying victimization | Unfair treatment by sexual orientation or gender identity | Unfair treatment by disability | 1.000 | Unfair treatment by sexual orientation or gender identity→ Unfair treatment by disability | 0.515 |
| Bullying victimization | Unfair treatment by race | Bullying victimization | 1.000 | Bullying victimization→ Unfair treatment by race | 0.525 |
| Bullying victimization | Unfair treatment by race | Unfair treatment by disability | 1.000 | Unfair treatment by disability→ Unfair treatment by race | 0.825 |
| Bullying victimization | Externalizing problems | Bullying victimization | 1.000 | Bullying victimization→ Externalizing problems | 0.865 |
| Bullying victimization | Unfair treatment by sexual orientation or gender identity | Externalizing problems | 0.995 | Unfair treatment by sexual orientation or gender identity→ Externalizing problems | 0.779 |
| Bullying victimization | Internalizing problems | Externalizing problems | 0.995 | Externalizing problems→ Internalizing problems | 0.563 |
| Bullying victimization | Unfair treatment by race | Unfair treatment by sexual orientation or gender identity | 0.975 | Unfair treatment by sexual orientation or gender identity→ Unfair treatment by race | 0.790 |
| Bullying victimization | Unfair treatment by sexual orientation or gender identity | Internalizing problems | 0.875 | Unfair treatment by sexual orientation or gender identity→ Internalizing problems | 0.646 |
| Bullying victimization | Internalizing problems | Bullying victimization | 0.785 | Bullying victimization→ Internalizing problems | 0.503 |
| Bullying victimization | Unfair treatment by disability | Bullying victimization | 0.700 | Bullying victimization→ Unfair treatment by disability | 0.607 |
| Bullying victimization | Unfair treatment by race | Internalizing problems | 0.270 | Internalizing problems→ Unfair treatment by race | 0.685 |
| Bullying perpetration | Externalizing problems | Bullying perpetration | 1.000 | Externalizing problems→ Bullying perpetration | 0.640 |
| Bullying perpetration | Internalizing problems | Externalizing problems | 1.000 | Internalizing problems→ Externalizing problems | 0.525 |
| Bullying perpetration | Unfair treatment by sexual orientation or gender identity | Externalizing problems | 1.000 | Unfair treatment by sexual orientation or gender identity→ Externalizing problems | 0.500 |
| Bullying perpetration | Unfair treatment by disability | Internalizing problems | 1.000 | Internalizing problems→Unfair treatment by disability | 0.615 |
| Bullying perpetration | Unfair treatment by race | Unfair treatment by disability | 1.000 | Unfair treatment by disability→ Unfair treatment by race | 0.750 |
| Bullying perpetration | Unfair treatment by sexual orientation or gender identity | Internalizing problems | 0.990 | Internalizing problems→ Unfair treatment by sexual orientation or gender identity | 0.646 |
| Bullying perpetration | Unfair treatment by sexual orientation or gender identity | Unfair treatment by disability | 0.975 | Unfair treatment by sexual orientation or gender identity→ Unfair treatment by disability | 0.544 |
| Bullying perpetration | Unfair treatment by race | Unfair treatment by sexual orientation or gender identity | 0.955 | Unfair treatment by sexual orientation or gender identity→ Unfair treatment by race | 0.780 |
| Bullying perpetration | Internalizing problems | Bullying perpetration | 0.930 | Internalizing problems -> Bullying perpetration | 0.629 |
| Bullying perpetration | Unfair treatment by sexual orientation or gender identity | Bullying perpetration | 0.880 | Unfair treatment by sexual orientation or gender identity→ Bullying perpetration | 0.699 |
| Bullying perpetration | Unfair treatment by race | Bullying perpetration | 0.700 | Bullying perpetration → Unfair treatment by race | 0.886 |
| Bullying perpetration | Unfair treatment by race | Internalizing problems | 0.350 | Unfair treatment by race → Internalizing problems | 0.543 |

Note. Edge presence probabilities summarize how often a connection appeared across weighted resamples of the focal-node graph. Directional support values indicate which orientation was more compatible with the resampled data.

**Supplementary Note 5. Expanded narrative summary of the exploratory overlap analyses.**

This note provides the fuller description of the exploratory overlap analyses. Because the three unfair-treatment indicators can co-occur within the same youth, we conducted supplementary weighted interaction analyses and SHAP-based interaction decompositions to explore whether overlap across domains mattered empirically beyond the additive contrasts emphasized in the main models. These exploratory analyses suggested that the highest predicted burden on both bullying victimization and bullying perpetration occurred under triple exposure, consistent with cumulative disadvantage across multiple unfair-treatment domains. At the same time, several pairwise interaction terms were negative, indicating that co-occurrence was not uniformly additive on the observed outcome scale and that combinations of exposures should not be read as a simple sum of single-domain coefficients. Because the predictive fit of the exploratory perpetration interaction model was limited, we interpret these findings as descriptive evidence that overlap matters, not as a definitive inter-sectional causal model. Tables 13-15 and Figures 7-9 should therefore be read as exploratory complements to the primary models rather than as replacements for them.

**Supplementary Table 13. Weighted model performance and interaction term estimates.**

| **Panel** | **Outcome** | **Measure or term** | **Statistic** | **95% CI** | ***p*** |
| --- | --- | --- | --- | --- | --- |
| Panel A |  | Model performance |  |  |  |
|  | Bullying victimization | Weighted RMSE | 0.825 |  |  |
|  | Bullying victimization | Weighted R-squared | 0.113 |  |  |
|  | Bullying perpetration | Weighted RMSE | 0.550 |  |  |
|  | Bullying perpetration | Weighted R-squared | 0.007 |  |  |
| Panel B |  | Weighted interaction term coefficients |  |  |  |
|  | Bullying victimization | Unfair treatment by race × Unfair treatment by sexual orientation or gender identity | -.195** | -.336, -.055 | 0.007 |
|  | Bullying victimization | Unfair treatment by race × Unfair treatment by disability | -.205* | -.383, -.027 | 0.024 |
|  | Bullying victimization | Unfair treatment by sexual orientation or gender identity × Unfair treatment by disability | -.582*** | -.780, -.383 | 0.000 |
|  | Bullying victimization | Unfair treatment by race × Unfair treatment by sexual orientation or gender identity × Unfair treatment by disability | .294 | -.031, .619 | 0.077 |
|  | Bullying perpetration | Unfair treatment by race × Unfair treatment by sexual orientation or gender identity | -.249*** | -.338, -.161 | 0.000 |
|  | Bullying perpetration | Unfair treatment by race × Unfair treatment by disability | .022 | -.090, .134 | 0.697 |
|  | Bullying perpetration | Unfair treatment by sexual orientation or gender identity × Unfair treatment by disability | -.033 | -.158, .092 | 0.600 |
|  | Bullying perpetration | Unfair treatment by race × Unfair treatment by sexual orientation or gender identity × Unfair treatment by disability | .357*** | .152, .561 | 0.001 |

Note. Panel A reports weighted predictive performance. Panel B reports weighted interaction term coefficients on the original outcome scales, with 95% confidence intervals in adjacent columns. The outward naming convention is retained for comparability with the manuscript.

**p* < 0.05, ***p* < 0.01, ****p* < 0.001.

**Supplementary Table 14. Top pairwise interaction intensities among the three unfair-treatment domains.**

| **Outcome** | **Exposure A** | **Exposure B** | **Mean absolute SHAP interaction** |
| --- | --- | --- | --- |
| Bullying victimization | Unfair treatment by sexual orientation or gender identity | Unfair treatment by disability | 0.002 |
| Bullying victimization | Unfair treatment by race | Unfair treatment by sexual orientation or gender identity | 0.001 |
| Bullying victimization | Unfair treatment by race | Unfair treatment by disability | 0.000 |
| Bullying perpetration | Unfair treatment by race | Unfair treatment by sexual orientation or gender identity | 0.001 |
| Bullying perpetration | Unfair treatment by race | Unfair treatment by disability | 0.000 |
| Bullying perpetration | Unfair treatment by sexual orientation or gender identity | Unfair treatment by disability | 0.000 |

Note. Mean absolute SHAP interaction values summarize the average strength with which each pair of unfair-treatment domains contributed jointly to the fitted weighted prediction models. Larger values indicate more prominent interaction structure.

**Supplementary Table 15. Weighted predicted outcome means across all eight exposure combinations.**

| **Outcome** | **Rank** | **Exposure combination** | **Weighted predicted mean** |
| --- | --- | --- | --- |
| Bullying victimization | 1 | UTR=1, UTSO=1, UTD=1 | 2.947 |
| Bullying victimization | 2 | UTR=1, UTSO=1, UTD=0 | 2.873 |
| Bullying victimization | 3 | UTR=0, UTSO=1, UTD=1 | 2.763 |
| Bullying victimization | 4 | UTR=0, UTSO=1, UTD=0 | 2.645 |
| Bullying victimization | 5 | UTR=1, UTSO=0, UTD=1 | 2.185 |
| Bullying victimization | 6 | UTR=0, UTSO=0, UTD=1 | 1.871 |
| Bullying victimization | 7 | UTR=1, UTSO=0, UTD=0 | 1.867 |
| Bullying victimization | 8 | UTR=0, UTSO=0, UTD=0 | 1.494 |
| Bullying perpetration | 1 | UTR=1, UTSO=1, UTD=1 | 1.563 |
| Bullying perpetration | 2 | UTR=0, UTSO=1, UTD=1 | 1.517 |
| Bullying perpetration | 3 | UTR=0, UTSO=1, UTD=0 | 1.499 |
| Bullying perpetration | 4 | UTR=1, UTSO=1, UTD=0 | 1.483 |
| Bullying perpetration | 5 | UTR=1, UTSO=0, UTD=1 | 1.308 |
| Bullying perpetration | 6 | UTR=1, UTSO=0, UTD=0 | 1.264 |
| Bullying perpetration | 7 | UTR=0, UTSO=0, UTD=1 | 1.168 |
| Bullying perpetration | 8 | UTR=0, UTSO=0, UTD=0 | 1.166 |

Note. Weighted predicted means were derived from the fitted weighted tree-ensemble models after setting the three unfair-treatment domains to each of the eight possible configurations. Higher values denote greater expected burden on the corresponding outcome.
